# Shorter versus longer antibiotic therapy for children with acute otitis media: A systematic review

**DOI:** 10.1007/s00431-026-06788-8

**Published:** 2026-02-28

**Authors:** Alexander Benkendorff, Sibylle Puntscher, Magdalena Flatscher-Thöni, Nikolai Mühlberger, Anne Göhner, Susanne Beck, Anneke Clara Bergt, Laura Krabbe, Thomas L. Heise, Lea Gorenflo, Siegbert Rieg, Markus Hufnagel, Claudia Breuer, Angela M. Kunzler, Uwe Siebert, Christine Schmucker, Joerg J. Meerpohl

**Affiliations:** 1 Institute for Evidence in Medicine, Medical Center - University of Freiburg / Medical Faculty - University of Freiburg, Freiburg, Germany; 2 Institute of Public Health, Medical Decision Making and Health Technology Assessment, Department of Public Health, Health Services Research and Health Technology Assessment, UMIT TIROL – University for Health Sciences and Technology, Hall in Tirol, Austria; 3 Planning, Control & Coordination Unit, District Office of Lörrach, Lörrach, Germany; 4 Chair of Criminal Law, Criminal Procedure Law, Comparative Criminal Law and Legal Philosophy, Leibniz University Hannover, Hannover, Germany; 5 Institute for Quality and Efficiency in Health Care (IQWiG), Cologne, Germany; 6 Cochrane Germany, Cochrane Germany Foundation, Freiburg, Germany; 7 Division of Infectious Diseases, Department of Medicine II, University Medical Centre Freiburg, Freiburg, Germany; 8 Division of Pediatric Rheumatology and Clinical Infectious Diseases, Department of General Pediatrics Adolescent Medicine and Neonatology, Center for Pediatrics, Medical Center and Medical Faculty, University of Freiburg, Freiburg, Germany; 9 Departments of Health Policy & Management and Epidemiology, Center for Health Decision Science, Harvard T.H. Chan School of Public Health, Boston, MA USA; 10 Institute for Technology Assessment and Department of Radiology, Massachusetts General Hospital, Harvard Medical School, Boston, MA USA

**Keywords:** Acute otitis media, Antibiotic therapy, Duration of therapy, Children, Systematic review

## Abstract

Overuse of antibiotics increases the risk of side effects and microbial resistance and leads to rising healthcare costs. Acute otitis media (AOM) is the leading cause of antibiotic prescription amongst children. However, recommendations differ regarding the duration of therapy. This systematic review compared short-course versus long-course antibiotic therapy on treatment outcomes of children with AOM and examined further implications of different treatment durations. To evaluate clinical efficacy, we performed systematic searches in MEDLINE, Embase, and CENTRAL (until 5 February 2024), searched trial registries and screened reference lists of systematic reviews including health technology assessment (HTA) reports. Randomized controlled trials (RCTs) comparing short-course and long-course antibiotic therapies in children with AOM were included. Non-inferiority was assumed for differences of less than 10%. Twelve RCTs investigating different antibiotic agents with a total of 3 409 children were included – mostly conducted before the year 2000 and mostly with a high risk of bias. No comparisons of short-course and long-course antibiotic therapies met the criteria for non-inferiority for treatment success; five studies even showed statistically significantly worse results with a shorter duration of therapy. Due to high statistical uncertainty, no statistically significant difference but also no non-inferiority could be demonstrated for relapse, mortality, or side effects. Poorer efficacy of shorter treatment durations may lead to higher costs for the healthcare system.

*Conclusion*: It cannot generally be assumed that a shorter antibiotic therapy leads to equivalent treatment results in children with AOM. However, new high-quality RCTs investigating treatment duration of commonly used antibiotics are highly needed. Shorter therapies offer economic benefits only when they are clinically as effective as longer ones.

*Registration:* PROSPERO (CRD42024519113).

**Table:** 

**What is Known:** • *Shorter antibiotic courses are recommended to reduce adverse effects, antimicrobial resistance, and costs.* • *For acute otitis media (AOM), a leading cause for antibiotic prescription in children, the optimal treatment duration remains controversial.*
**What is New:** • *This systematic review, including data from twelve RCTs, found no evidence of non-inferiority for shorter antibiotic therapies in pediatric AOM. Some studies for some antibiotic agents even showed poorer outcomes of shorter treatment durations. If not equally effective, shorter treatment may also increase costs for the healthcare system.* • *Additional high-quality RCTs investigating commonly prescribed antibiotic substances like amoxicillin are needed.*

**Supplementary Information:**

The online version contains supplementary material available at 10.1007/s00431-026-06788-8.

## Introduction

The use of antibiotics causes costs to the healthcare system and can lead to environmental problems [1–4]. Furthermore, excessive and inappropriate use of antibiotics promotes antimicrobial resistance, which is a growing global health issue [5]. While previously, sufficiently long antibiotic therapy was recommended to prevent antimicrobial resistance [6], within the scientific community the “mantra” has shifted towards "shorter is better" [7–9]. This shift was based on findings that every administration of antibiotics can cause side effects [9] or induce antimicrobial resistance [8].

With approximately 709 million cases worldwide each year, acute otitis media (AOM) is one of the most common infectious diseases and the most frequent reason for doctor visits and prescriptions of antibiotics in children [10, 11]. Children under the age of 6 are particularly affected [12, 13], usually in combination with an upper respiratory tract infection [14]. The swollen nasopharyngeal mucosa and consequently impaired ventilation of the middle ear allow bacteria from the local flora, such as *Streptococcus pneumoniae*, *Haemophilus influenzae,* or *Moraxella catarrhalis,* to ascend and cause infection [14, 15].

Since viruses are initially detectable in a large proportion of AOM cases [16, 17], initial therapy focuses on symptoms and does not always require antibiotic treatment [18–21]. If antibiotic therapy is deemed necessary, amoxicillin is the first choice [18–21]. National guidelines recommend treatment durations between 5 and 10 days, varying by guideline commission and sometimes by age-specific recommendations [18–21]. However, there are currently no high-quality systematic reviews on the optimal duration of antibiotic therapy for AOM.

The aim of this systematic review was to compare the efficacy and safety of different antibiotic treatment durations for oral antibiotic treatment in children with AOM. In addition, non-medical effects of shorter antibiotic treatment for AOM were investigated, including economic, environmental, and social aspects.

## Methods

This publication is based on a Health Technology Assessment (HTA) report (ThemenCheck report) [22] commissioned by the Institute for Quality and Efficiency in Health Care (IQWiG). Besides AOM, this report also evaluated the duration of antibiotic therapy for community-acquired pneumonia (CAP) in children and adults. However, due to differences in populations and therapies, these two infections were evaluated separately after a combined search for studies. This work focuses exclusively on results relating to AOM. This review was conducted after registration in PROSPERO (CRD42024519113) and in accordance with the PRISMA (Preferred Reporting Items for Systematic reviews and Meta-Analyses) standards [23].

The electronic databases MEDLINE, CENTRAL, and Embase were searched on 6 February 2024, followed by searches in four study registries (ClinicalTrials.gov, International Clinical Trials Registry Platform, EU Clinical Trials Register, Clinical Trials Information System) and reference lists checks of relevant systematic reviews, including HTA reports. We included randomized controlled trials (RCTs) that compared different durations of oral antibiotic therapy with the same drug at the same dosage in children with AOM in an outpatient setting. Two independent reviewers (ACB, LG, or CS) screened each record on title and abstract level for potential relevance and later reviewed potentially relevant records in full text. Discrepancies were resolved by discussion or by consulting the third reviewer.

From the included studies, we extracted data on study characteristics, population characteristics, and outcomes into standardized tables. The main outcomes examined were treatment success (individual study definitions of this outcome are provided in Table S1), recurrence of infection, mortality, adverse events (AEs, including hospitalizations), and quality of life, as well as the additional outcomes adherence, antimicrobial resistance, and bacterial eradication. Data extraction was carried out by one reviewer and checked by a second reviewer (AB, ACB, or CS). Discrepancies were resolved by discussion or by consulting the third reviewer. Risk of bias was assessed according to IQWiG methods [24], with an assessment on study level evaluating the appropriate generation of the randomization sequence, allocation concealment, blinding of patient(s) and treating staff, and results-independent reporting, as well as additional aspects. An additional outcome-specific assessment included blinding of outcome assessors, implementation of the intention-to-treat (ITT) principle, and results-independent reporting, as well as additional aspects. Risk of bias was evaluated independently by two reviewers (AB and ACB). Discrepancies were resolved by discussion or consultation of a third reviewer (CS).

The results were summarized in systematic evidence tables and, where possible, evaluated on the basis of relative risks (RR) with 95% confidence intervals (CI) using meta-analyses. Based on recommendations from the European Medicines Agency (EMA), a 10% difference was considered an acceptable non-inferiority margin [25]. Accordingly, non-inferiority was assumed, if the RR with 95%-CI did not exceed the non-inferiority margin of 0.9 for favorable outcomes (treatment success) or 1.1 for negative outcomes (recurrence, mortality, AE). Otherwise, no indication of non-inferiority was inferred. However, this does not automatically imply inferiority of the shorter treatment regimen.

Detailed information on the methods can be found in the *Supplementary Methods* in the *Appendix a*nd in the ThemenCheck report protocol [26].

## Results

### Search

**Fig. 1:**
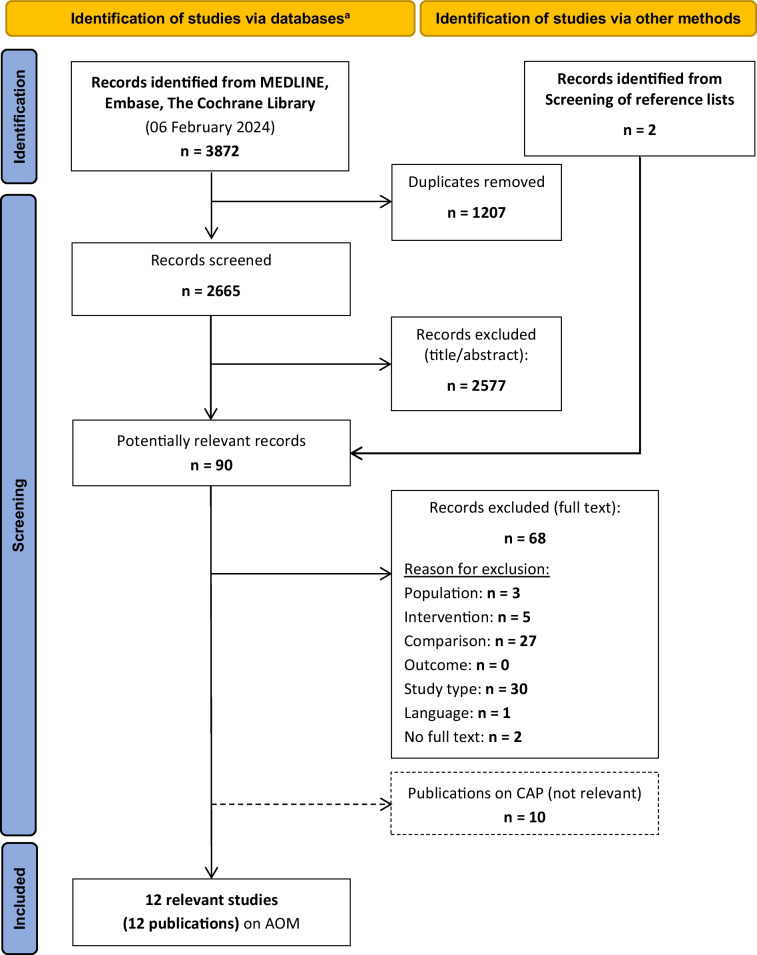
Study selection according to the PRISMA flow diagram. AOM: Acute otitis media; CAP: Community-acquired pneumonia; n: number; PRISMA: Preferred Reporting Items for Systematic Reviews and Meta-Analyses

Figure 1 shows the study selection process. Of 3 874 hits, twelve studies with a total of 3 409 children were included [27–38].

### Study characteristics

Table 1 shows the main characteristics of the included studies, stratified by antibiotic agent and duration of treatment. The antibiotics penicillin V, amoxicillin, amoxicillin clavulanic acid, a first-generation cephalosporin (cefaclor), a second-generation cephalosporin (cefuroxime), and third-generation cephalosporins (cefixime and cefpodoxime) were investigated. Treatment durations of 2 vs. 7 days, 3 vs. 7 days, 3 vs. 10 days, 5 vs. 10 days, and 10 vs. 20 days were compared. The studies were conducted in Europe or North America and, with the exception of one study [34], conducted before the year 2000. On study level, only one study was classified as having a low risk of bias [34] and eleven studies as having a high risk of bias [27–33, 35–38] (Table S2). The high risk of bias was mainly due to a lack of information on the randomization sequence generation or concealment of group allocation, as well as the absence of a study protocol/study registry entry. For the outcomes of treatment success and recurrence, one study was excluded from the analysis irrespective of available data due to an excessive number of study dropouts (> 30%) [31].

**Table 1:** Characteristics of the included studies

Study [reference]	Study design	No. of children^a^	Age of children	Dosage of antibiotic	Location and time period	Investigated Outcomes
**2 vs. 7 days Penicillin V**						
** Meistrup-Larsen (1983) **[38]	RCT	101	1–10 years	55 mg/kg/day	Denmark, 11/1980–05/1981	Treatment success, recurrence
**5 vs. 10 days Penicillin V**						
** Ingvarsson (1982)**^**b **^[35]	RCT^c^	134	6 months – 7 years	50 mg/kg/day	Sweden, 12/1976–04/1977	Treatment success, recurrence^d^, AE
**3 vs. 10 days Amoxicillin**						
** Chaput de Saintonge (1982) **[28]	RCT	84	2–10 years	375 mg/day (< 5 years) 750 mg/day (> 5 years)	United Kingdom, 1979–1980	Treatment success, recurrence; mortality, AE
**10 vs. 20 days Amoxicillin**						
** Mandel (1995) **[37]	RCT	181^e,f^	7 months – 12 years	40 mg/kg/day	USA, 12/1987–12/1990	Recurrence^d^, AE
**5 vs. 10 days Amoxicillin-clavulanic acid**						
** Hoberman (1997) **[33]	RCT^c^	580^e^	2 months – 12 years	45/6.4 mg/kg/day	USA/Canada, 01/1994–07/1994	Treatment success^d^, recurrence, AE
** Cohen (1998) **[30]	RCT	385	< 30 months	80/10 mg/kg/day	France, 11/1994–06/1996	Treatment success^d^, recurrence, AE
** Hoberman (2016) **[34]	RCT	515	6–23 months	90/6.4 mg/kg/day	United States, 01/2012–09/2015	Treatment success, recurrence, AE
**3 vs. 7 days First-generation cephalosporin (Cefaclor)**						
** Jones (1986) **[36]	RCT	100^f^	3–10 years	375 mg/day	United Kingdom, 1983–1985	Recurrence, mortality
**5 vs. 10 days First-generation cephalosporin (Cefaclor)**						
** Hendrickse (1988) **[32]	RCT	175	1 month – 12 years	40 mg/kg/day	USA, Duration: unclear	Treatment success, recurrence^d^, AE
**5 vs. 10 days Second-generation cephalosporin (Cefuroxime)**						
** Gooch (1996) **[31]	RCT	477	3 months – 12 years	30 mg/kg/day	USA, Duration: unclear	AE^g^
**5 vs. 10 days Third-generation cephalosporins (Cefixime, Cefpodoxime)**						
** Adam (2000) **[27]	RCT	227	2–14 years	8 mg/kg/day (cefixime)	Germany, 03/1999–09/1999	Treatment success, recurrence; AE
** Cohen (2000) **[29]	RCT	450	< 30 months	8 mg/kg/day (cefpodoxime)	France, 10/1996–04/1997	Treatment success^d^, recurrence; AE

*mg/kg/day* milligrams per kilogram of body weight per day, *No.* Number, *RCT* randomized controlled trial, *AE* adverse events

^a ^The number of randomized children per study is indicated

^b ^In Ingvarsson (1982), only the second phase of the study could be taken into account, as only in this phase both groups received the same antibiotic dosage

^c^ The studies by Ingvarsson (1982) and Hoberman (1997) were the only studies that were not placebo-controlled

^d^ This outcome was reported at several time points

^e^ A third study arm was not included because it did not meet the inclusion criteria

^f^ For 19 (< 20%) of the included children in the relevant study arms, it remains unclear whether treatment was provided on an outpatient or inpatient basis

^g^ Although treatment success and recurrence were also reported, these outcomes were not included in the analysis due to too many study dropouts (> 30% for each outcome)

### Treatment success

Nine studies reported data on the outcome treatment success, one of which had a low [34] and eight of which had a high outcome-specific risk of bias [27–30, 32, 33, 35, 38] (Table S3). Non-inferiority could not be demonstrated for any of the comparisons examined. For most comparisons, the 95% CI of the RR (pooled or from individual studies) crossed the non-inferiority margin of 0.9. In Hoberman et al. (2016), which compared 5 vs. 10 days of amoxicillin-clavulanic acid, the 95% CI was even completely below the non-inferiority margin of the RR of 0.9 (RR [95% CI]: 0.79 [0.71; 0.88]) and showed a statistically significantly worse result for the shorter antibiotic therapy duration. Further comparisons (5 vs. 10 days of amoxicillin-clavulanic acid, 5 vs. 10 days of cefaclor, 5 vs. 10 days of third-generation cephalosporins) also showed statistically significantly worse results for shorter antibiotic therapy duration at the primary evaluation time point. The results of the most important comparisons are presented in Table 2, with further results in Table 3. Forest plots of comparisons with more than one study are shown in Figs. S1-S3.

**Table 2:** Results for the most important comparisons and time points

Outcome	Events with longer therapy^a^	Events with shorter therapy	Absolute difference^b^ of shorter therapy compared to longer therapy [95% CI]	Relative Risk [95% CI]	Study participants^c^ (studies)	Comment
**3 vs. 10 days Amoxicillin**						
Treatment success (day 13–16)	929 per 1000	881 per 1000	48 fewer per 1000 [162 fewer to 84 more]	0.95 [0.83; 1.09]	84 (1 study) [28]	unclear^d^
Recurrence (up to 18 months)^e^	167 per 1000	190 per 1000	24 more per 1000 [91 fewer to 311 more]	1.14 [0.46; 2.87]	84 (1 study) [28]	unclear^d^
**5 vs. 10 days Amoxicillin-clavulanic acid**						
Treatment success (day 12–14)	Meta-analysis not informative due to high statistical uncertainty, no pooling (Figure S1)					**Statistically significantly poorer effect**
Hoberman (1997)	840 per 1000	747 per 1000	**92 fewer per 1000** **[152 to 27 fewer]**	**0.89** **[0.82; 0.97]**	580 (1 study) [33]	
Cohen (1998)	849 per 1000	734 per 1000	**115 fewer per 1000** **[188 to 34 fewer]**	**0.86** **[0.78; 0.96]**	378 (1 study) [30]	
Hoberman (2016)	836 per 1000	664 per 1000	**172 fewer per 1000** **[240 to 97 fewer]**	**0.79** **[0.71; 0.88]**	467 (1 study) [34]	
Recurrence	Meta-analysis not informative due to high statistical uncertainty, no pooling (Figure S4)					unclear^d^
Hoberman (1997) (days 10 to 38)	178 per 1000	109 per 1000	**68 fewer per 1000** **[105 to 13 fewer]**	**0.61** **[0.41; 0.93]**	580 (1 study) [33]	
Cohen (1998)^f^ (days 14 to 42)	201 per 1000	142 per 1000	60 fewer per 1000 [117 fewer to 38 more]	0.70 [0.42; 1.19]	280 (1 study) [30]	
Hoberman (2016) (entire season)	433 per 1000	393 per 1000	40 fewer per 1000 [118 fewer to 56 more]	0.91 [0.73; 1.13]	457 (1 study) [34]	
**3 vs. 7 days First-generation Cephalosporin (Cefaclor)**						
Treatment success	No usable data reported					-
Recurrence (days 7 to 42)	157 per 1000	178 per 1000	21 more per 1000 [84 fewer to 278 more]	1.13 [0.46; 2.77]	96 (1 study) [36]	unclear^d^
**5 vs. 10 days First-generation cephalosporin (Cefaclor)**						
Treatment success (day 10)	935 per 1000	811 per 1000	**124 fewer per 1000 [219 to 16 fewer]**	**0.87** **[0.77; 0.98]**	151 (1 study) [32]	**Statistically significant poorer effect**
Recurrence (day 14 to 30)	150 per 1000	125 per 1000	25 fewer per 1000 [102 fewer to 177 more]	0.83 [0.32; 2.18]	108 (1 study) [32]	unclear^d^
**5 vs. 10 days Third-generation cephalosporins (Cefixime, Cefpodoxime)**						
Treatment success (day 11–14)	922 per 1000	835 per 1000	**84 fewer per 1000 [131 to 35 fewer]**	**0.91** **[0.86; 0.96]**	660 (2 studies, Figure S3) [27, 29]	**Statistically significantly poorer effect**
Recurrence (day 1 to 42)	163 per 1000	146 per 1000	16 fewer per 1000 [75 fewer to 81 more]	0.90 [0.54; 1.49]	329 (1 study) [29]	unclear^d^

*CI* confidence interval, *RR* relative risk, statistically significant results are highlighted in **bold**

^a^ The baseline risk was calculated as the sum of events in the control group divided by the number of study participants evaluated in the control group. Together with the relative risk (RR), this served as the basis for calculating the frequency of events in the shorter therapy group and the absolute difference with confidence interval (CI). Differences in baseline risk can be explained by differences in the underlying studies, particularly in terms of inclusion criteria, definitions of treatment success and timing of outcome assessment

^b^ The absolute difference with CI was calculated using GRADEpro [65]. Rounding may result in deviations

^c^ Only the evaluated study participants (after exclusions) are displayed

^d^ Non-inferiority could not be shown as the non-inferiority margin was exceeded. However, the lack of evidence of non-inferiority does not imply inferiority of the shorter antibiotic therapy

^e^ The evaluation period varied and ranged up to 18 months after the start of therapy. The median period was 12 months

^f^ Only children with successful treatment on days 12–14 were considered for this outcome by Cohen (1998)

**Table 3:** Results for further comparisons and other time points

Outcome	Events with longer therapy^a^	Events with shorter therapy	Absolute difference^b^ of shorter therapy compared to longer therapy [95% CI]	Relative Risk [95% CI]	Study participants^c^ (studies)	Comment
**2 vs. 7 days Penicillin V**						
Treatment success (day 1–7)	764 per 1000	717 per 1000	46 fewer per 1000 [196 fewer to 142 more]	0.94 [0.74; 1.19]	101 (1 study) [38]	unclear^d^
Recurrence (up to day 14)	55 per 1000	109 per 1000	54 more per 1000 [27 fewer to 376 more]	1.99 [0.50; 7.90]	101 (1 study) [38]	unclear^d^
Mortality	No data reported					
**5 vs. 10 days Penicillin V**						
Treatment success (day 28—30)	853 per 1000	864 per 1000	11 more per 1000 [100 fewer to 138 more]	1.01 [0.88; 1.16]	134 (1 study) [35]	unclear^d^
Recurrence (up to 1 month)	59 per 1000	61 per 1000	2 more per 1000 [43 fewer to 174 more]	1.03 [0.27; 3.95]	134 (1 study) [35]	unclear^d^
Recurrence (up to 6 months)	279 per 1000	303 per 1000	24 more per 1000 [101 fewer to 235 more]	1.08 [0.64; 1.84]	134 (1 study) [35]	unclear^d^
Mortality	No data reported					
**3 vs. 10 days Amoxicillin**						
Mortality	0 per 1000	0 per 1000	-	-	84 (1 study) [28]	unclear^d^
**10 vs. 20 days Amoxicillin**						
Treatment success	No usable data reported					
Recurrence (up to day 20)	34 per 1000	54 per 1000	20 more per 1000 [21 fewer to 184 more]	1.58 [0.39; 6.40]	181 (1 study) [37]	unclear^d^
Recurrence (up to day 90)	388 per 1000	453 per 1000	65 more per 1000 [70 fewer to 258 more]	1.17 [0.82; 1.66]	171 (1 study) [37]	unclear^d^
Mortality	No data reported					
**5 vs. 10 days Amoxicillin-clavulanic acid**						
Treatment success (days 28–42)	593 per 1000	563 per 1000	30 fewer per 1000 [88 fewer to 34 more]	0.95 [0.85; 1.06]	955 (2 studies, Figure S2) [30, 33]	unclear^d^
Mortality	No data reported					
**3 vs. 7 days First-generation cephalosporin (Cefaclor)**						
Mortality	0 per 1000	0 per 1000	-	-	98 (1 study) [36]	unclear^d^
**5 vs. 10 days First-generation cephalosporin (Cefaclor)**						
Recurrence (day 10 to 13)	14 per 1000	6 per 100	8 fewer per 1000 [14 fewer to 120 more]	0.40 [0.02; 9.63]	132 (1 study) [32]	unclear^d^
Recurrence (day 31 to 60)	113 per 1000	98 per 100	16 fewer per 1000 [84 fewer to 210 more]	0.86 [0.26; 2.85]	94 (1 study) [32]	unclear^d^
Recurrence (day 61 to 90)	82 per 1000	53 per 1000	29 fewer per 1000 [71 fewer to 191 more]	0.64 [0.12; 3.34]	87 (1 study) [32]	unclear^d^
Mortality	No data reported					
**5 vs. 10 days Second-generation cephalosporin (Cefuroxime)**						
Treatment success (day 24–28)	No data reported					
Recurrence (day 14 to 28)	No data reported					
Mortality	No data reported					
**5 vs. 10 days Third-generation cephalosporins (Cefixime, Cefpodoxime)**						
Treatment success (day 28–42)	671 per 1000	624 per 1000	47 fewer per 1000 [127 fewer to 44 more]	0.93 [0.81; 1.07]	448 (1 study) [29]	unclear^d^
Mortality	No data reported					

*CI* confidence interval, *RR* relative risk

^a^ The baseline risk was calculated as the sum of events in the control group divided by the number of study participants evaluated in the control group. Together with the relative risk (RR), this served as the basis for calculating the frequency of events in the shorter therapy group and the absolute difference with confidence interval (CI). Differences in baseline risk can be explained by differences in the underlying studies, particularly in terms of inclusion criteria, definitions of treatment success and timing of outcome assessment

^b^ The absolute difference with CI was calculated using GRADEpro [65]. Rounding may result in deviations

^c^ Only the evaluated study participants (after exclusions) are displayed

^d^ Non-inferiority could not be shown as the non-inferiority margin was exceeded. However, the lack of evidence of non-inferiority does not imply inferiority of the shorter antibiotic therapy

### Recurrence

Ten studies reported data on the outcome recurrence. All had a high outcome-specific risk of bias [28–30, 32–38] (Table S3). The results had a high statistical uncertainty, which manifested in wide confidence intervals. Therefore, no non-inferiority of the shorter treatment duration was demonstrated for any comparison (Tables 2 and 3, Figure S4). However, results did not show a significant difference in recurrence rates between the treatment regimens either.

### Mortality

No study reported on the outcome mortality in a structured way. However, in two studies, data on AE suggested that no deaths had occurred (Table 3). Both had a high risk of bias (Table S3) and investigated 3 vs. 10 days of amoxicillin [28] and 3 vs. 7 days of cefaclor [36].

### Adverse events

Eight studies reported usable data on AEs – one with a low risk of bias [34] and seven with a high risk of bias [27–31, 33, 37] (Table S3). Non-inferiority could not be established for any comparison due to high statistical uncertainty in most comparisons (Table S4, Figure S5-S7). When comparing 10 vs. 20 days of amoxicillin, there were even statistically significantly more AEs in the shorter treatment group from day 11 to 20 [37]. However, 20 days of antibiotic treatment would be an unusually long treatment duration for AOM [18–21].

### Quality of life

None of the included studies provided information on health-related quality of life.

### Additional outcomes

Adherence was reported comparatively in four studies. In the three placebo-controlled studies [30, 31, 37], adherence did not differ statistically significantly between the groups. Here, both groups were exposed to the intervention/placebo for the same length of time. In contrast, in a non-placebo-controlled study comparing 5 vs. 10 days of amoxicillin clavulanic acid [33], adherence was statistically significantly higher in the group receiving the shorter course of antibiotic therapy (Table S5).

Four studies provided data on microbiological outcomes [30, 31, 34, 35]. Only in one study [30] a slight difference between the treatment groups occurred in positive bacterial detection and detection of resistant bacteria after the end of treatment (Tables S6-S7).

### Economic considerations

We identified one health economic modelling study on AOM [39]. The study investigated amoxicillin in children aged 6 months to 12 years in the context of the US healthcare system over an analytical time horizon of 30 days. Using an incremental cost utility approach the study compared a treatment duration of 5 days with 7 to 10 days and found higher costs for a shorter treatment duration [39]. Due to poorer efficacy, the higher costs for parental absence from work and doctor visits outweighed the lower immediate drug costs. A treatment duration of 7 to 10 days was determined to be the most efficient option with the highest utility value. However, another strategy "delayed prescription" (prescribing antibiotics only if symptoms persist after 48 to 72 h), was identified as the most cost-effective strategy [39].

### Environmental aspects

During production, use, or disposal, antibiotic metabolites can contaminate the environment and promote antimicrobial resistance [40–43]. Additionally, in countries with high average income per capita, the healthcare sector—in particular the production and transport of medicines—contribute significantly to carbon dioxide (CO_2_) emissions [1, 4]. The production of 1 g of amoxicillin causes emissions of about 14.3 g of carbon dioxide [4]. The use of (high dose) antibiotic tablets instead of antibiotic suspensions can reduce CO_2_ emission per gram antibiotics [44]. Also, shorter antibiotic treatment courses may reduce CO_2_ emissions and environmental impacts by reducing demand [2]. However, if treatments are less effective and require subsequent treatment, the environmental impact could ultimately be higher.

### Social aspects

In AOM, there is an association between socio-economic disadvantage, higher probability of illness and therapy regimen employed [29, 30, 45–48]. Furthermore, hearing impairments due to middle ear effusion as well as frequent relapses can result in negative consequences for children's education and development [9, 49–54]. However, antibiotic therapy only targets the infection and does not directly affect the middle ear effusion, which causes the hearing impairment [53].

## Discussion

For AOM in children, no comparison of different treatment durations showed non-inferiority of shorter antibiotic treatment courses in terms of treatment success. For the antibiotics amoxicillin-clavulanic acid, cefaclor and third-generation cephalosporins, shorter treatment durations even showed statistically significantly worse results. For the antibiotics penicillin V and amoxicillin, the results for treatment success showed no statistically significant difference between the groups, but due to high statistical uncertainty non-inferiority could not be demonstrated either. Likewise, for other outcomes (recurrence, mortality, AE), non-inferiority could not be demonstrated due to high statistical uncertainty. Ultimately, poorer efficacy can lead to increased absenteeism at work among parents and guardians and more visits to the doctor, resulting in higher treatment costs. Only adherence to therapy may be higher with shorter treatment durations.

In this review, only studies with the same oral antibiotic agent in the same dosage were considered. Previous systematic reviews [55, 56], which also included comparisons of different antibiotics and parenteral antibiotic therapies, similarly showed poorer efficacy for shorter antibiotic therapies – with the exception of azithromycin and ceftriaxone. A systematic review with network meta-analysis [57], which did not report non-inferiority margins, found a non-inferiority of a 7-day treatment course compared to a 10-day treatment course with amoxicillin and amoxicillin-clavulanic acid. This was based on indirect comparisons. Therefore, further trials are needed investigating common treatment duration for frequently prescribed antibiotic agents, e.g. 5/7 days of amoxicillin vs. 10 days of amoxicillin.

Given the breadth of search methods used, relevant bias due to publication bias appears unlikely. However, within the included studies, only one study had a low risk of bias, while all other studies had a high risk of bias, limiting the certainty of evidence. Due to the study periods (many studies were conducted before 2000) and different study locations, differences in the resistance spectrum of bacteria are possible. Especially in high-income countries, vaccination with pneumococcal conjugate vaccines and frequent antibiotic treatment changed pneumococcal serotypes and resistance patterns [58]. This may limit both comparability of the studies and transferability of the study results to today's clinical practice. Despite dynamic changes, *Streptococcus pneumoniae*, *Haemophilus influenzae,* and *Moraxella catarrhalis* continue to be the most common bacterial causes for AOM [13, 59].

In the included studies, antimicrobial resistance was only investigated in a few studies. In addition, treatment success was defined differently in some cases and external effects of shortened antibiotic therapy were not investigated. When investigating non-inferiority, the uncritical use of ITT analysis may also lead to bias [60]. For that reason, per-protocol (PP) analysis was also considered in this review (where available), but always provided comparable results.

Many guidelines now recommend antibiotic treatment durations tailored to age, for example, 10 days for children under 2 years of age, 7 days for children aged 2 to 6 years, and 5 to 7 days for children older than 6 years [20, 61]. Although some of the included studies observed higher rates of treatment failure and recurrence in younger children overall, they rarely provided stratified data for comparison of treatment durations in different age groups [28, 29, 32, 33, 35, 37]. Only one study [33] observed less difference in treatment regimens with increasing age of the included children.

Furthermore, not all children in all of the included studies met the indications for recommended antibiotic therapy according to current guidelines [18–21]. In addition, in this as well as in other systematic reviews [55, 56], only few studies were identified that investigated amoxicillin as the first-line drug in children with AOM. Likewise, few studies included older children or adolescents.

In this study, evaluation of outcomes was based on relative effects, which may differ in absolute effects depending on the frequency of events (absolute effects in Tables 2 and 3). For that reason, in the overall assessment across the investigated outcomes, highest priority should be given to the most frequent and most relevant outcomes – in particular treatment success. However, no equivalent efficacy could be demonstrated for this outcome in particular. For medical decision-making and guideline development, efficacy outcomes should be weighed up against side effects using formal benefit-risk analyses [62, 63] and, if necessary, decision-analytic modelling [64]. This study provides the basis for future analyses, guideline development, and medical decision making. Given the limitations of the underlying studies, this review also identifies research gaps with necessity for future clinical trials as well as uncertainties in evidence that should be acknowledged and communicated by healthcare professionals and taken into account in (shared) medical decision making.

In conclusion, it cannot be generally assumed that shorter antibiotic treatment regimens have a comparable effect to longer antibiotic therapy courses in children with AOM. While absence of non-inferiority does not automatically imply inferiority, in some studies for some antibiotic agents however, the treatment success rate with shorter treatment duration was even statistically significantly lower. No advantages in terms of adverse events or bacterial resistance could be demonstrated, while in some cases even higher overall costs were observed. Only adherence may increase with shorter antibiotic treatment courses. However, most included studies were conducted before the year 2000 and had a high risk of bias. Furthermore, analyses of different age groups and up-to-date high-quality studies on the use of common antibiotics, like amoxicillin, according to current indications are lacking. This underscores the need for new high-quality RCTs in this field.

## Supplementary Information

Below is the link to the electronic supplementary material.

Supplementary file1 (DOCX 256 KB)

## Data Availability

All collected data on included studies are available in the ThemenCheck report T23-04 "Antibiotic therapy: Does a shorter duration of administration lead to comparable treatment results?" [22].

## Abbreviations

AE: Adverse event
AOM: Acute otitis media
CAP: Community-acquired pneumonia
CI: Confidence interval
CO_2_: Carbon dioxide
HTA: Health technology assessment
IQWiG: Institute for Quality and Efficiency in Health Care
ITT: Intention-to-treat
PRISMA: Preferred Reporting Items for Systematic reviews and Meta-Analyses
RCT: Randomized controlled trial
RR: Relative risk
