# Supplementary material for: Shorter versus longer antibiotic therapy for children with acute otitis media: A systematic review
Source: Eur J Pediatr. 2026 Feb 28;185(3):155. doi: 10.1007/s00431-026-06788-8 (PMC12950071; doi:10.1007/s00431-026-06788-8)
Supplement: Supplementary file 1 — Supplementary file1 (DOCX 256 KB) [file 431_2026_6788_MOESM1_ESM.docx]

**Appendix**

[**Definition of the outcome "treatment success" in the included studies 2**](#_Toc208496843)

[**Assessment of risk of bias 4**](#_Toc208496844)

[**Supplementary results 7**](#_Toc208496845)

[*Treatment success* 7](#_Toc208496846)

[*Recurrence* 8](#_Toc208496847)

[*Adverse Events* 9](#_Toc208496848)

[*Adherence* 12](#_Toc208496849)

[*Microbiological outcomes* 13](#_Toc208496850)

[**Supplementary methods 16**](#_Toc208496851)

[Search strategies 20](#_Toc208496855)

[List of screened systematic reviews 33](#_Toc208496856)

[List of excluded studies (on full text level) 36](#_Toc208496857)

## **Definition of the outcome "treatment success" in the included studies**

| Table S1: Definition of the outcome "treatment success" for acute otitis media (AOM) | |
| --- | --- |
| **Study** | **Definition of treatment success** |
| **Acute otitis media – children** | |
| **2 vs. 7 days of penicillin V** | |
| **Meistrup-Larsen (1983)** | Defined as patient not crying from pain after day one, analgesics unnecessary after day one, no otitis symptoms after day two, otorrhea for less than six days, no contralateral otitis during first week. |
| **5 vs. 10 days of penicillin V** | |
| **Ingvarsson (1982)** | By day 30; defined as normal eardrums with normal mobility tested with a pneumatic otoscope and, in cases that could be investigated, normal hearing AND absence of treatment failure (insufficient effect of therapy or a new attack of otitis during antibiotic therapy, relapse) |
| **3 vs. 10 days of amoxicillin** | |
| **Chaput de Saintonge (1982)** | By day 13-16; defined as absence of treatment failure (defined as recovery rate rated as unsatisfactory by general practitioner AND additional antibiotic treatment required; measured by otalgia, hearing loss, nasal congestion, cough or fever, appearance of eardrum) |
| **10 vs. 20 days of amoxicillin** | |
| **Mandel (1995)** | Only reported for day 10 (not relevant for this review) |
| **5 vs. 10 days of amoxicillin-clavulanic acid** | |
| **Hoberman (1997)** | By day 12-14: defined as complete resolution or improvement of symptoms AND no further antibiotic therapy necessary  By day 32-38: defined as complete resolution or improvement of symptoms AND no further antibiotic therapy necessary |
| **Cohen (1998)** | By day 12-14; defined as absence or significant improvement of symptoms (fever, otalgia, irritability, otoscopic signs of AOM) AND no relapse or complication or need for further antibiotic therapy or tympanocentesis  By day 28-42; defined as successful treatment by day 14 AND no recurrence AND no persistent AOM with effusion AND no need for further antibiotic therapy |
| **Hoberman (2016)** | By day 12-14; defined as absence of treatment failure (defined as worsening of symptoms or of otoscopic findings or incomplete resolution of symptoms attributable to acute otitis media) |
| **3 vs. 7 days of first-generation cephalosporin (cefaclor)** | |
| **Jones (1986)** | By day 7; defined as reduction in symptoms and number of days with symptoms; measured by tympanic membrane signs, earache, nasal discharge, cough, waking up crying |
| **5 vs. 10 days of first-generation cephalosporin (cefaclor)** | |
| **Hendrickse (1988)** | By day 14: defined as no relapse AND no reinfection AND elimination of middle ear effusion or eardrum healing without other symptoms or persistent effusion with absence of erythematous bulging tympanic membrane, fever and pain |
| **5 vs. 10 days of second-generation cephalosporin (cefuroxime)** | |
| **Gooch (1996)** | By day 24-28; defined as response to treatment by day 3-5 AND absence of recurrence AND healing (defined as absence or improvement of symptoms, including otoscopic findings on day 11-14 AND absence of symptoms, including middle ear effusion, on day 24-28) OR improvement (defined as significant improvement in symptoms OR persistent middle ear effusion with absence of other symptoms on day 24-28) |
| **5 vs. 10 days of third-generation cephalosporins (cefixime, cefpodoxime)** | |
| **Adam (2000)** | By day 11; defined as complete recovery or improvement of symptoms |
| **Cohen (2000)** | By day 12-14; defined as absence or significant improvement in symptoms (measured by fever, otalgia, irritability, otoscopic signs of AOM) AND no relapse or complications or need for further antibiotic therapy or tympanic membrane perforation  By day 28-42; defined as treatment success by day 14 AND no recurrence AND no persistent AOM with effusion AND no further antibiotic therapy |

## **Assessment of risk of bias**

| Table S2: Risk of bias on study level | | | | | | | |
| --- | --- | --- | --- | --- | --- | --- | --- |
| Studie | Adequate generation of the randomisation sequence | Concealment of group assignment | Blinding | | Independent reporting of results | Absence of other aspects | Risk of bias on study level |
|  |  |  | Participant | Treating staff |  |  |  |
| **2 vs. 7 days Penicillin V** | | | | | | | |
| Meistrup-Larsen (1983) | unclear^a^ | unclear^b^ | yes | yes | no^c,d^ | no^e^ | **high** |
| **5 vs. 10 days Penicillin V** | | | | | | | |
| Ingvarsson (1982) | unclear^a^ | unclear^b^ | no^f^ | no^f^ | unclear^c^ | yes | **high** |
| **3 vs. 10 days Amoxicillin** | | | | | | | |
| Chaput de Saintonge (1982) | unclear^a^ | unclear^b^ | yes | yes | unclear^c^ | yes | **high** |
| **10 vs. 20 days Amoxicillin** | | | | | | | |
| Mandel (1995) | unclear^a^ | unclear^b^ | yes | yes | unclear^c^ | yes^g^ | **high** |
| **5 vs. 10 days Amoxicillin-clavulanic acid** | | | | | | | |
| Hoberman (1997) | unclear^a^ | unclear^b^ | no^f^ | yes | unclear^c^ | yes | **high** |
| Cohen (1998) | yes | yes | yes | yes | unclear^c^ | unclear^h^ | **high** |
| Hoberman (2016) | unclear^a^ | yes | yes | yes | yes | unclear^i^ | **low** |
| **3 vs. 7 days First-generation cephalosporin (Cefaclor)** | | | | | | | |
| Jones (1986) | unclear^a^ | unclear^b^ | yes | yes | unclear^c^ | unclear^h^ | **high** |
| **5 vs. 10 days First-generation cephalosporin (Cefaclor)** | | | | | | | |
| Hendrickse (1988) | yes | unclear^b^ | yes | yes | unclear^c^ | unclear^h^ | **high** |
| **5 vs. 10 days Second-generation cephalosporin (Cefuroxime)** | | | | | | | |
| Gooch (1996) | unclear^a^ | unclear^b^ | yes | yes | unclear^c^ | unclear^h^ | **high** |
| **5 vs. 10 days Third-generation cephalosporins (Cefixime, Cefpodoxime)** | | | | | | | |
| Adam (2000) | unclear^a^ | unclear^b^ | yes | yes | no^c,j^ | unclear^h^ | **high** |
| Cohen (2000) | yes | yes | yes | yes | unclear^c^ | unclear^h^ | **high** |
| Risk of bias was assessed according to the methods of the Institute for Quality and Efficiency in Health Care (IQWiG) [5]  a. The studies are described as randomized, but information on allocation sequence generation is missing.  b. The studies did not provide enough information on allocation concealment.  c. No information on planned outcomes (no registry entry or study protocol).  d. The primary outcome is only defined in the results section without further explanation.  e. Contradictory information on study group sizes and dropouts.  f. No information on blinding and no placebo used.  g. Early termination of studies due to lack of financial support.  h. Study dropouts and/or reasons for dropping out are not reported separately for each group.  i. Early termination of studies due to success at interim analysis but no further details provided.  j. Reporting of secondary outcomes missing without explanation. | | | | | | | |

| Table S3: Outcome-specific risk of bias | | | | | | |
| --- | --- | --- | --- | --- | --- | --- |
| **Study** | **Risk of bias on study level** | **Blinding of outcome assessor** | **Implementation of intention-to-treat principle^a^** | **Independent reporting of outcome** | **Absence of other aspects** | **Outcome-specific risk of bias** |
| **2 vs. 7 days Penicillin V** | | | | | | |
| Meistrup-Larsen (1983) | |  |  |  |  |  |
| Treatment success | **high** | yes | yes | no^b,c^ | yes | **high** |
| Recurrence | **high** | unclear | unclear | unclear^b^ | yes | **high** |
| **5 vs. 10 days Penicillin V** | | | | | | |
| Ingvarsson (1982) |  |  |  |  |  |  |
| Treatment success | **high** | no | yes | unclear^b^ | yes | **high** |
| Recurrence | **high** | no | yes | unclear^b^ | yes | **high** |
| **3 vs. 10 days Amoxicillin** | | | | | | |
| Chaput de Saintonge (1982) | |  |  |  |  |  |
| Treatment success | **high** | yes | yes | unclear^b^ | yes | **high** |
| Recurrence | **high** | unclear | yes | unclear^b^ | yes | **high** |
| Mortality | **high** | yes^d^ | yes | unclear^b^ | yes | **high** |
| Adverse Events | **high** | yes | yes | unclear^b^ | yes | **high** |
| **10 vs. 20 days Amoxicillin** | | | | | | |
| Mandel (1995) |  |  |  |  |  |  |
| Recurrence | **high** | unclear | yes | unclear^b^ | yes | **high** |
| Adverse Events | **high** | unclear | no | unclear^b^ | yes | **high** |
| **5 vs. 10 days Amoxicillin-clavulanic acid** | | | | | | |
| Hoberman (1997) |  |  |  |  |  |  |
| Treatment success | **high** | no | yes | unclear^b^ | yes | **high** |
| Recurrence | **high** | no | yes | unclear^b^ | yes | **high** |
| Adverse Events | **high** | no | yes | unclear^b^ | yes | **high** |
| Cohen (1998) |  |  |  |  |  |  |
| Treatment success | **high** | yes | yes | unclear^b^ | yes | **high** |
| Recurrence | **high** | yes | yes | unclear^b^ | yes | **high** |
| Adverse Events | **high** | yes | yes | unclear^b^ | no^e^ | **high** |
| Hoberman (2016) |  |  |  |  |  |  |
| Treatment success | **low** | yes | yes | yes | unclear^f^ | **low** |
| Recurrence | **low** | yes | no | unclear^g^ | yes | **high** |
| Adverse Events | **low** | yes | yes | yes | yes | **low** |
| **3 vs. 7 days First-generation cephalosporin (Cefaclor)** | | | | | | |
| Jones (1986) |  |  |  |  |  |  |
| Recurrence | **high** | unclear | unclear | unclear^b^ | yes | **high** |
| Mortality | **high** | yes^d^ | unclear | unclear^b^ | yes | **high** |
| **5 vs. 10 days First-generation cephalosporin (Cefaclor)** | | | | | | |
| Hendrickse (1988) | |  |  |  |  |  |
| Treatment success | **high** | unclear | no | unclear^b^ | yes | **high** |
| Recurrence | **high** | unclear | no | unclear^b^ | yes | **high** |
| **5 vs. 10 days Second-generation cephalosporin (Cefuroxime)** | | | | | | |
| Gooch (1996) |  |  |  |  |  |  |
| Treatment success | **high** | yes | no | unclear^b^ | yes | **high** |
| Recurrence | **high** | yes | no | unclear^b^ | yes | **high** |
| Adverse Events | **high** | yes | yes | unclear^b^ | yes | **high** |
| **5 vs. 10 days Third-generation cephalosporins (Cefixime, Cefpodoxime)** | | | | | | |
| Adam (2000) |  |  |  |  |  |  |
| Treatment success | **high** | unclear | no | unclear^b^ | yes | **high** |
| Adverse Events | **high** | unclear | yes | unclear^b^ | yes | **high** |
| Cohen (2000) |  |  |  |  |  |  |
| Treatment success | **high** | yes | yes | unclear^b^ | yes | **high** |
| Recurrence | **high** | yes | yes | unclear^b^ | yes | **high** |
| Adverse Events | **high** | yes | yes | unclear^b^ | yes | **high** |
| Risk of bias was assessed according to the methods of the Institute for Quality and Efficiency in Health Care (IQWiG) [5]  a. Implementation of ITT-principle was considered adequate if more than 90% of the randomized participants were included in the analysis and the difference between the groups was less than 5%.  b. No information on planned outcomes (no registry entry or study protocol).  c. Unusual definition of outcome without further explanation.  d. For the outcome of mortality, blinding was considered irrelevant and therefore rated with “yes”.  e. There are already statistically significant differences at baseline concerning the AE diarrhea.  f. Early termination of study due to success at interim analysis but no further details provided.  g. Only one of two planned time points is reported. | | | | | | |

##

## **Supplementary results**

***Treatment success***


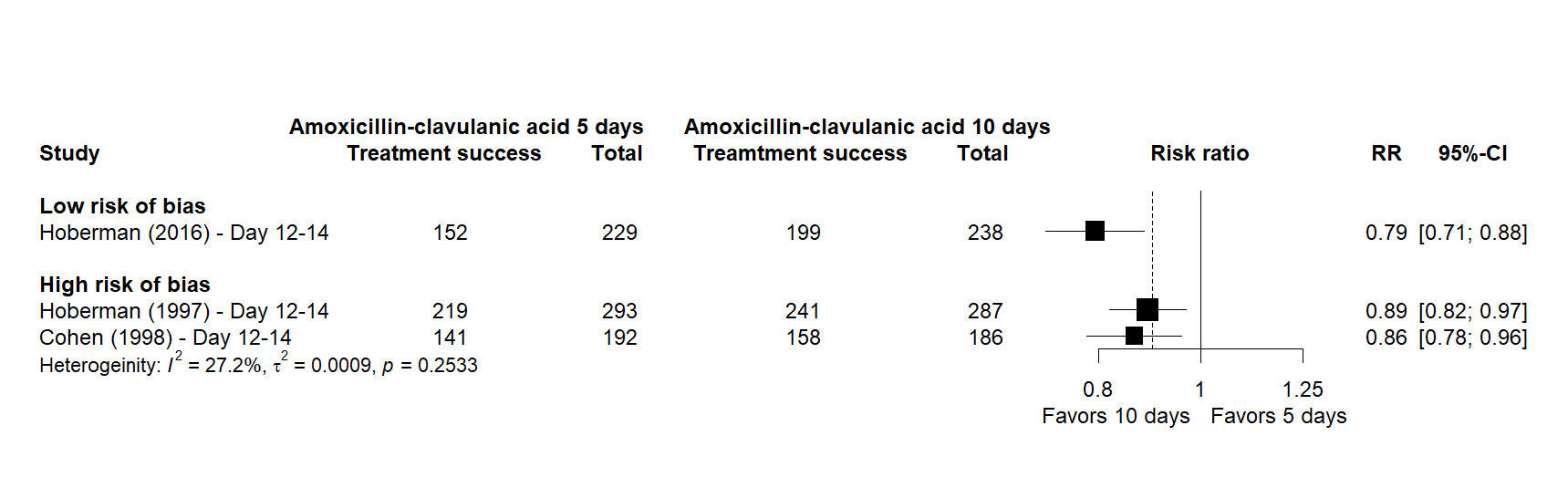


Figure S1: Meta-analysis for the outcome Treatment success for 5 vs. 10 days Amoxicillin-clavulanic acid (primary time point)


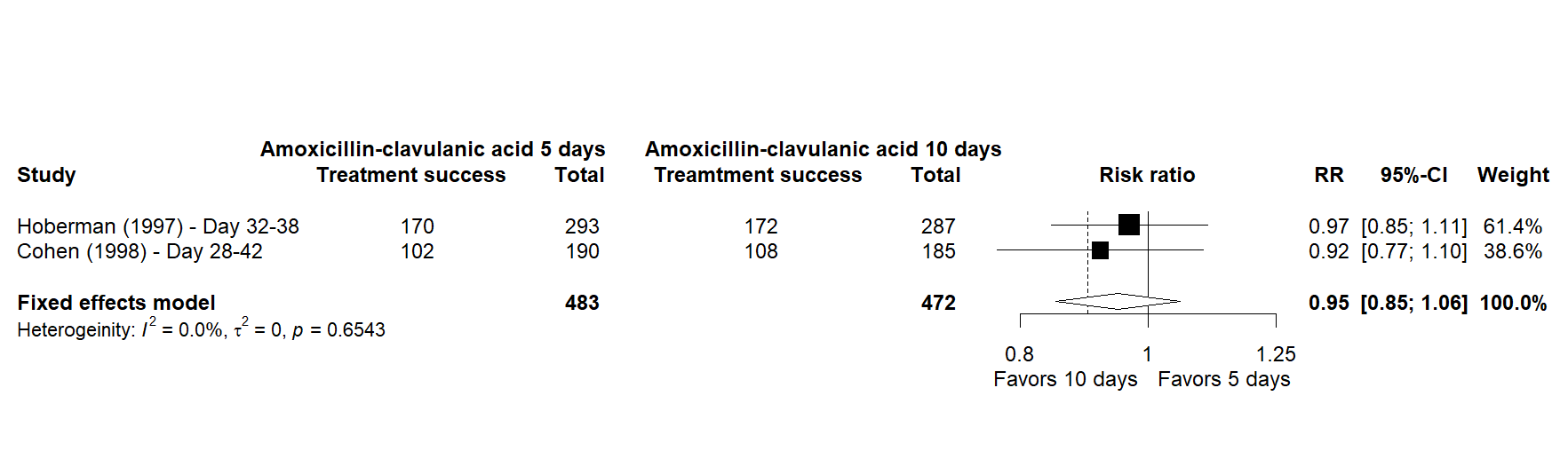


Figure S2: Meta-analysis for the outcome Treatment success for 5 vs. 10 days Amoxicillin-clavulanic acid (later time point)


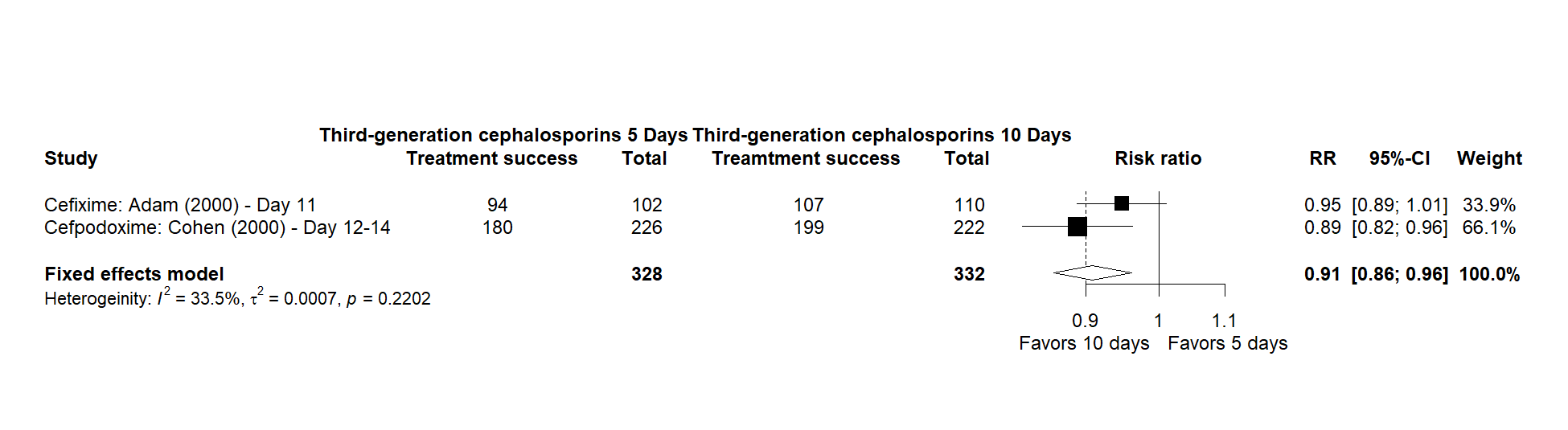


Figure S3: Meta-analysis for the outcome Treatment success for 5 vs. 10 days Third-generation cephalosporins (Cefixime, Cefpodoxime) (primary time point)

### ***Recurrence***


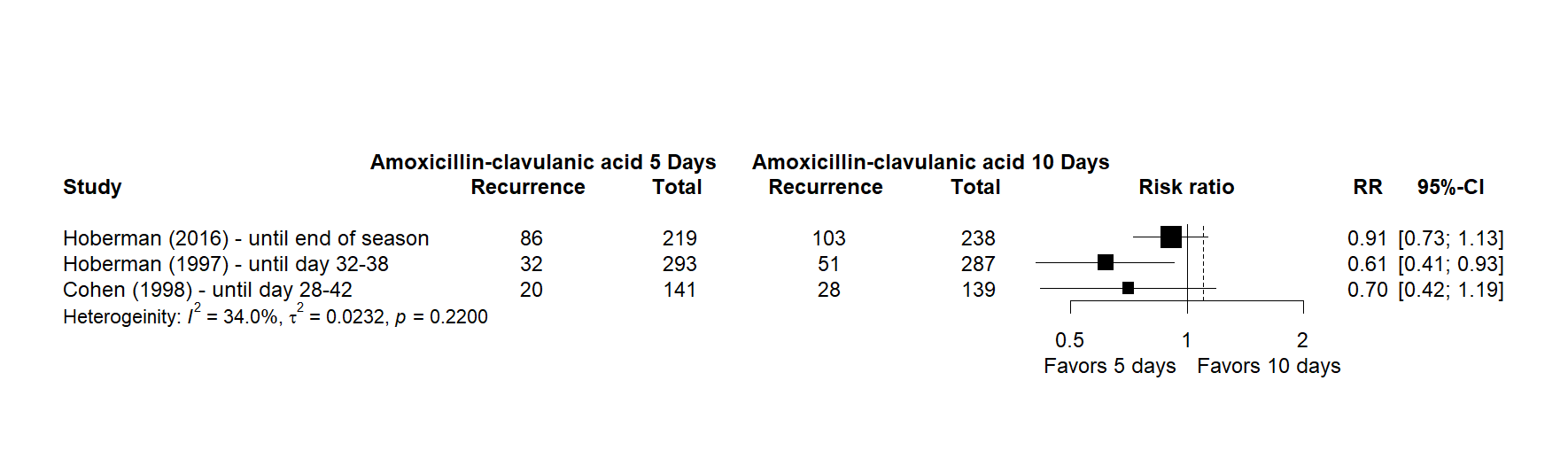


Figure S4: Meta-analysis for the outcome Recurrence with 5 vs. 10 days Amoxicillin-clavulanic acid

### ***Adverse Events***

| Table S4: Adverse events in included studies | | | | | | | | | | | | |
| --- | --- | --- | --- | --- | --- | --- | --- | --- | --- | --- | --- | --- |
| **Study**  **Type of AE**  **Time span** | **Shorter Therapy** | | | |  | **Longer Therapy** | | |  | **Shorter vs. Longer Therapy** | | |
|  | **Analyzed Participants** | | **Participants with the AE** | |  | **Analyzed Participants** | **Participants with the AE** | |  | **Risk ratio** | **[95 %-CI]** | **p-Value** |
|  |  |  | **n** | **%** |  |  | **n** | **%** |  |  |  |  |
| **2 vs. 7 days Penicillin V** | | | | | | | | | | | | |
| **Meistrup-Larsen (1983)** | | No information on AE. | | | | | | | | | | |
| **5 vs. 10 days Penicillin V** | | | | | | | | | | | | |
| **Ingvarsson (1982)** | Reporting of AE only summarized for all study groups and study phases. | | | | | | | | | | | |
| **3 vs. 10 days Amoxicillin** | | | | | | | | | | | | |
| **Chaput de Saintonge (1982)** | | | | | | | | | | | | |
| Day 1 – Day 10^a^ | 42 | | 4 | 9.5 |  | 42 | 1 | 2.4 |  | 4.00 | [0.47; 34.31] | 0.206 |
| **10 vs. 20 days Amoxicillin** | | | | | | | | | | | | |
| **Mandel (1995)** | | | | | | | | | | | | |
| Day 11 – Day 20 | 103^b^ | | 11 | 10.7 |  | 100^b^ | 2 | 2.0 |  | **5.34** | **[1.21; 23.49]** | 0.027 |
| - *Diarrhea* | *103^b^* | | *4* | *3.9* |  | *100^b^* | *0* | *0.0* |  | *8.74* | *[0.48; 160.23]* | *0.144* |
| - *Nausea and/or vomiting* | *103^b^* | | *3* | *2.9* |  | *100^b^* | *0* | *0.0* |  | *6.80* | *[0.36; 129.92]* | *0.203* |
| - *Rash/ hypersensitivity* | *103^b^* | | *2* | *1.9* |  | *100^b^* | *1* | *1.0* |  | *1.94* | *[0.18; 21.08]* | *0.585* |
| - *Lack of appetite* | *103^b^* | | *2* | *1.9* |  | *100^b^* | *0* | *0.0* |  | *4.86* | *[0.24; 99.88]* | *0.306* |
| - *Constipation* | *103^b^* | | *0* | *0.0* |  | *100^b^* | *1* | *1.0* |  | *0.32* | *[0.01; 7.85]* | *0.488* |
| **5 vs. 10 days Amoxicillin-clavulanic acid** | | | | | | | | | | | | |
| **Hoberman (1997)** | | | | | | | | | | | | |
| Day 1 – Day 40 |  | |  |  |  |  |  |  |  |  |  |  |
| - *Diarrhea^c^* | *293* | | *32* | *10.9* |  | *287* | *35* | *12.2* |  | *0.90* | *[0.57; 1.41]* | *0.632* |
| - *Vomiting* | *293* | | *21* | *7.2* |  | *287* | *29* | *10.1* |  | *0.71* | *[0.41; 1.21]* | *0.210* |
| - *Diaper rash* | *293* | | *15* | *5.1* |  | *287* | *23* | *8.0* |  | *0.64* | *[0.34; 1.20]* | *0.163* |
| **Cohen (1998)** | | | | | | | | | | | | |
| Day 1 – Day 42 | 194 | | 60^d^ | 30.9 |  | 188 | 55^d^ | 29.3 |  | 1.06 | [0.78; 1.44] | 0.722 |
| - *Diarrhea* | *194* | | *44^d^* | *22.7* |  | *188* | *49^d^* | *26.1* |  | *0.87* | *[0.61; 1.24]* | *0.442* |
| - *Skin rash* | *194* | | *8^d^* | *4.1* |  | *188* | *13^d^* | *6.9* |  | *0.60* | *[0.25; 1.41]* | *0.237* |
| - *Withdrawal because of AE* | *194* | | *8^d^* | *4.1* |  | *188* | *4^d^* | *2.1* |  | *1.94* | *[0.59; 6.33]* | *0.273* |
| **Hoberman (2016)** | | | | | | | | | | | | |
| Day 1 – Day 16 |  | |  |  |  |  |  |  |  |  |  |  |
| - *Diarrhea* | *258* | | *75* | *29.1* |  | *257* | *78* | *30.4* |  | *0.96* | *[0.73; 1.25]* | *0.751* |
| - *Dermatitis* | *258* | | *87* | *33.7* |  | *257* | *85* | *33.1* |  | *1.02* | *[0.80; 1.30]* | *0.876* |
| **3 vs. 7 days First-generation cephalosporin (Cefaclor)** | | | | | | | | | | | | |
| **Jones (1986)** | No information on AE. | | | | | | | | | | | |
| **5 vs. 10 days First-generation cephalosporin (Cefaclor)** | | | | | | | | | | | | |
| **Hendrickse (1988)** | Reporting of AE only summarized for all study groups. | | | | | | | | | | | |
| **5 vs. 10 days Second-generation cephalosporin (Cefuroxime)** | | | | | | | | | | | | |
| **Gooch (1996)** | | | | | | | | | | | | |
| Day 1 – Day 28 | 242 | | 48^d^ | 19.8 |  | 235 | 37^d^ | 15.7 |  | 1.26 | [0.85; 1.86] | 0.245 |
| - *Diarrhea/ loose bowel movements* | *242* | | *28^d^* | *11.6* |  | *235* | *18^d^* | *7.7* |  | *1.51* | *[0.86; 2.66]* | *0.152* |
| - *Vomiting* | *242* | | *16^d^* | *6.6* |  | *235* | *9^d^* | *3.8* |  | *1.73* | *[0.78; 3.83]* | *0.179* |
| - *Other AE* | *242* | | *12^d^* | *5.0* |  | *235* | *17^d^* | *7.2* |  | *0.69* | *[0.33; 1.40]* | *0.302* |
| - *Withdrawal because of AE* | *242* | | *13^d^* | *5.4* |  | *235* | *8^d^* | *3.4* |  | *1.58* | *[0.67; 3.74]* | *0.300* |
| **5 vs. 10 days Third-generation cephalosporins (Cefixime, Cefpodoxime)** | | | | | | | | | | | | |
| **Adam (2000)** | | | | | | | | | | | | |
| Day 1 – Day 28 | 115 | | 8 | 7.0 |  | 113 | 4 | 3.5 |  | 1.97 | [0.61; 6.34] | 0.258 |
| - *Gastrointestinal disorders* | *115* | | *5* | *4.3* |  | *113* | *4* | *3.5* |  | *1.23* | *[0.34; 4.46]* | *0.755* |
| - *Psychiatric disorders* | *115* | | *1* | *0.9* |  | *113* | *0* | *0.0* |  | *2.95* | *[0.12; 71.61]* | *0.507* |
| - *Urinary system disorders* | *115* | | *1* | *0.9* |  | *113* | *0* | *0.0* |  | *2.95* | *[0.12; 71.61]* | *0.507* |
| - *Other* | *115* | | *1* | *0.9* |  | *113* | *0* | *0.0* |  | *2.95* | *[0.12; 71.61]* | *0.507* |
| **Cohen (2000)** | | | | | | | | | | | | |
| Day 1 – Day 28 | 224 | | 26 | 11.6 |  | 222 | 36 | 16.2 |  | 0.72 | [0.45; 1.14] | 0.162 |
| - *Withdrawal because of AE* | *224* | | *2* | *0.9* |  | *222* | *2* | *0.9* |  | *0.99* | *[0.14; 6.97]* | *0.993* |
| AE: adverse event; CI: Confidence interval  a. Chaput de Saintonge (1982) reports AE (especially diarrhea) leading to discontinuation of therapy.  b. Mandel (1995) reports AE for episodes of AOM and not on participant level.  c. Hoberman (1997) distinguishes protocol-defined diarrhea and withdrawals due to diarrhea. Both aspects had comparable results and are presented summarized.  d. Participants might experience more than one AE. | | | | | | | | | | | | |


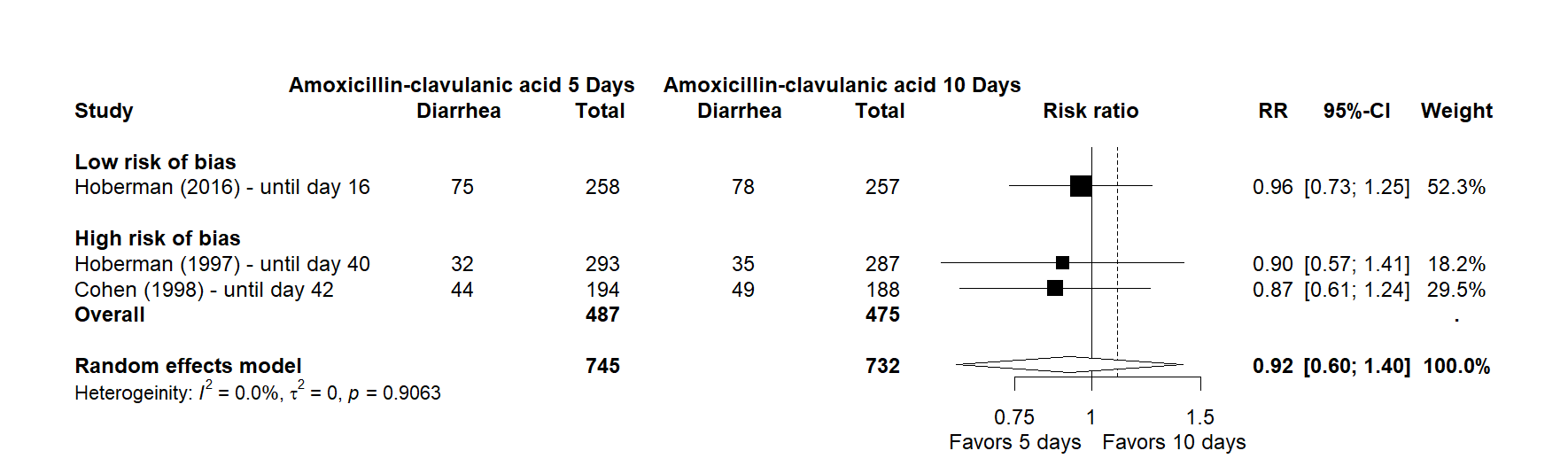


Figure S5: Meta-analysis for the outcome Diarrhea (AE) with 5 vs. 10 days Amoxicillin-clavulanic acid


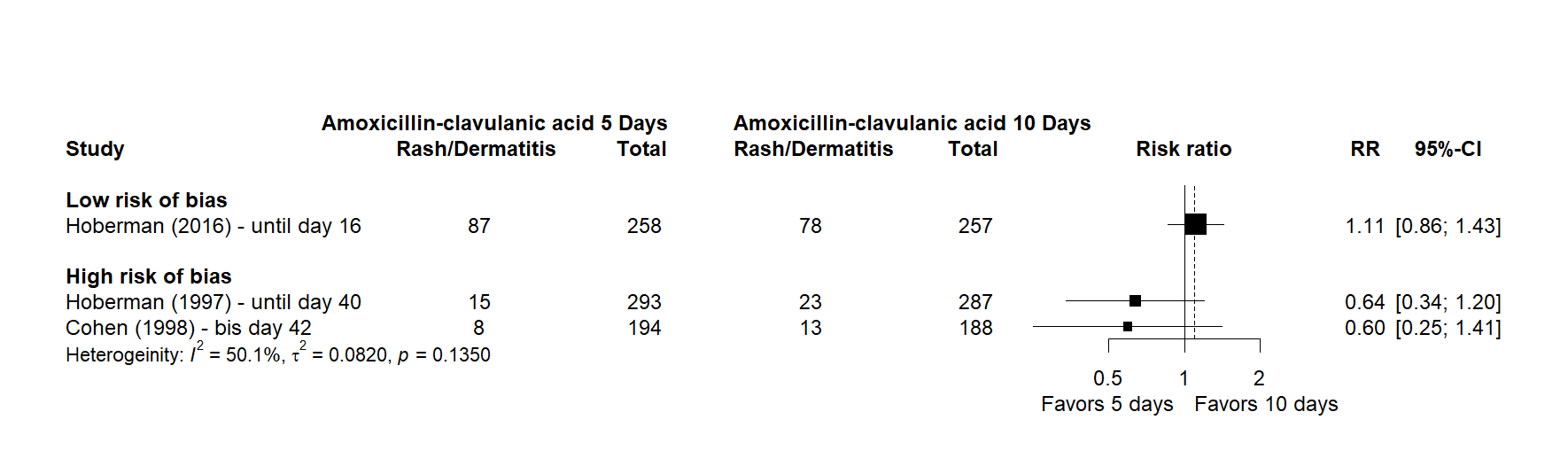


Figure S6: Meta-analysis for the outcome Rash/Dermatitis (AE) with 5 vs. 10 days Amoxicillin-clavulanic acid


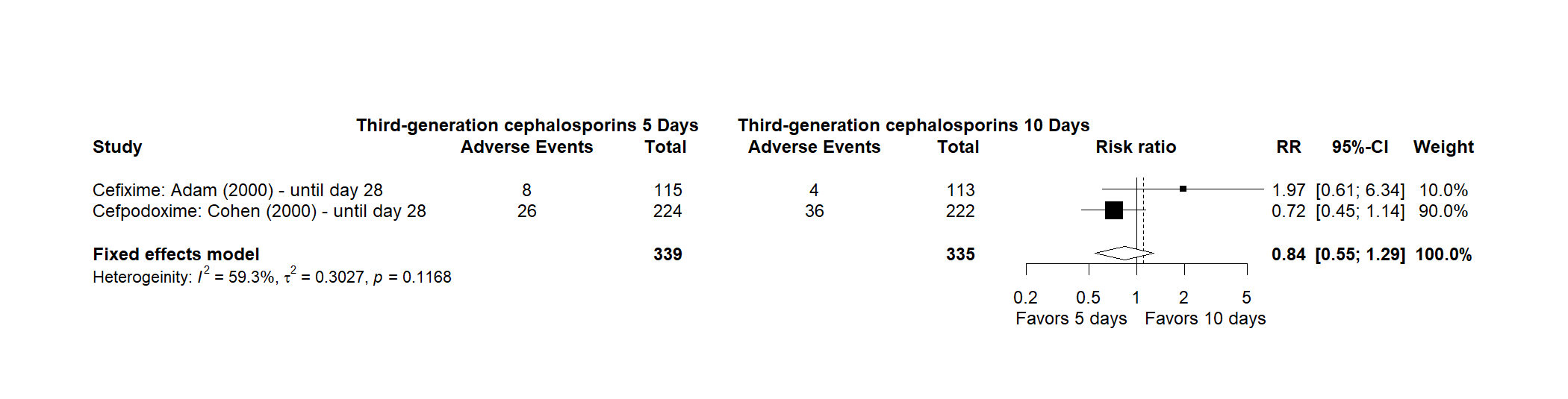


Figure S7: Meta-analysis for the outcome AE (overall) for 5 vs. 10 days Third-generation cephalosporins (Cefixime, Cefpodoxime)

### ***Adherence***

| Table S5: Adherence in included studies | | | | | | | | | | | | | |
| --- | --- | --- | --- | --- | --- | --- | --- | --- | --- | --- | --- | --- | --- |
| **Study**  **Time point** | **Shorter Therapy** | | | | |  | **Longer Therapy** | | |  | **Shorter vs. Longer Therapy** | | |
|  | **Analyzed Participants** | | | **Participants adhering** | |  | **Analyzed Participants** | **Participants adhering** | |  | **Risk ratio** | **[95 %-CI]** | **p-Value** |
|  |  |  |  | **n** | **%** |  |  | **n** | **%** |  |  |  |  |
| **2 vs. 7 days Penicillin V** | | | | | | | | | | | | | |
| **Meistrup-Larsen (1983)** | | No information on adherence. | | | | | | | | | | | |
| **5 vs. 10 days Penicillin V** | | | | | | | | | | | | | |
| **Ingvarsson (1982)** | | No information on adherence. | | | | | | | | | | | |
| **3 vs. 10 days Amoxicillin** | | | | | | | | | | | | | |
| **Chaput de Saintonge (1982)** | | | Reporting of adherence only summarized for all study groups. | | | | | | | | | | |
| **10 vs. 20 days Amoxicillin** | | | | | | | | | | | | | |
| **Mandel (1995)** | | | | | | | | | | | | | |
| Day 10 | 99.2% / 97.6% / 97.1%^a,b^ | | | | |  | 96.7% / 97.7% / 95.6%^a,b^ | | |  | Calculation not possible | | |
| Day 20 | 93.6% / 7.7% / 91.6%^a,b^ | | | | |  | 96.7% / 78.8% / 94.3%^a,b^ | | |  | Calculation not possible | | |
| **5 vs. 10 days Amoxicillin-clavulanic acid** | | | | | | | | | | | | | |
| **Hoberman (1997)** | | | | | | | | | | | | | |
| No information on time point | 293 | | | 284 | 96.9 |  | 287 | 257 | 89.6 |  | **1.08** | **[1.04; 1.13]** | <0.001 |
| **Cohen (1998)** | | | | | | | | | | | | | |
| No information on time point | 172 | | | 160 | 93.0 |  | 173 | 163 | 94.2 |  | 0.99 | [0.93; 1.04] | 0.649 |
| **Hoberman (2016)** | No information on adherence. | | | | | | | | | | | | |
| **3 vs. 7 days First-generation cephalosporin (Cefaclor)** | | | | | | | | | | | | | |
| **Jones (1986)** | No information on adherence. | | | | | | | | | | | | |
| **5 vs. 10 days First-generation cephalosporin (Cefaclor)** | | | | | | | | | | | | | |
| **Hendrickse (1988)** | Reporting of adherence only summarized for all study groups. | | | | | | | | | | | | |
| **5 vs. 10 days Second-generation cephalosporin (Cefuroxime)** | | | | | | | | | | | | | |
| **Gooch (1996)** | | | | | | | | | | | | | |
| Day 3, 5, 11-14 | 92.0%^a^ | | | | |  | 96.0%^a^ | | |  | Calculation not possible | | |
| **5 vs. 10 days Third-generation cephalosporins (Cefixime, Cefpodoxime)** | | | | | | | | | | | | | |
| **Adam (2000)** | No information on adherence. | | | | | | | | | | | | |
| **Cohen (2000)** | Reporting of adherence only summarized for all study groups. | | | | | | | | | | | | |
| CI: Confidence interval  a. This study only reports percentages of participants with adherence to therapy.  b. Mandel (1995) reports adherence according to medication returned, urine analysis, and diaries. | | | | | | | | | | | | | |

### ***Microbiological outcomes***

| Table S6: Resistance of bacteria against antibiotics in included studies | | | | | | | | | | | | |
| --- | --- | --- | --- | --- | --- | --- | --- | --- | --- | --- | --- | --- |
| **Study**  **Resistant bacteria**  **Time point** | **Shorter Therapy** | | | |  | **Longer Therapy** | | |  | **Shorter vs. Longer Therapy** | | |
|  | **Analyzed Participants** | | **Participants with resistant bacteria** | |  | **Analyzed Participants** | **Participants with resistant bacteria** | |  | **Risk ratio** | **[95 %-CI]** | **p-Value** |
|  |  |  | **n** | **%** |  |  | **n** | **%** |  |  |  |  |
| **2 vs. 7 days Penicillin V** | | | | | | | | | | | | |
| **Meistrup-Larsen (1983)** | | No information on microbiological outcomes. | | | | | | | | | | |
| **5 vs. 10 days Penicillin V** | | | | | | | | | | | | |
| **Ingvarsson (1982)** | | No information on resistance in bacteria. | | | | | | | | | | |
| **3 vs. 10 days Amoxicillin** | | | | | | | | | | | | |
| **Chaput de Saintonge (1982)** | | No information on microbiological outcomes. | | | | | | | | | | |
| **10 vs. 20 days Amoxicillin** | | | | | | | | | | | | |
| **Mandel (1995)** | | No information on microbiological outcomes. | | | | | | | | | | |
| **5 vs. 10 days Amoxicillin-clavulanic acid** | | | | | | | | | | | | |
| **Hoberman (1997)** | | No information on microbiological outcomes. | | | | | | | | | | |
| **Cohen (1998)** | | No information on microbiological outcomes. | | | | | | | | | | |
| **Hoberman (2016)** | | | | | | | | | | | | |
| New penicillin nonsusceptible bacteria strains  Day 12-14 | 177 | | 78 | 44.1 |  | 181 | 85 | 47.0 |  | 0.94 | [0.75; 1.18] | 0.583 |
| Share of penicillin nonsusceptible bacteria strains  Day 12-14 |  | |  |  |  |  |  |  |  |  |  |  |
| - *S. pneumoniae* | *36* | | *14* | *38.9* |  | *28* | *13* | *46.4* |  | *0.84* | *[0.47; 1.48]* | *0.543* |
| - *H. influenzae* | *65* | | *28* | *43.1* |  | *77* | *38* | *49.4* |  | *0.87* | *[0.61; 1.25]* | *0.459* |
| Share of penicillin nonsusceptible bacteria strains  End of Season |  | |  |  |  |  |  |  |  |  |  |  |
| - *S. pneumoniae* | *70* | | *11* | *15.7* |  | *88* | *9* | *10.2* |  | *1.54* | *[0.67; 3.50]* | *0.306* |
| - *H. influenzae* | *21* | | *7* | *33.3* |  | *25* | *9* | *36.0* |  | *0.93* | *[0.42; 2.06]* | *0.850* |
| **3 vs. 7 days First-generation cephalosporin (Cefaclor)** | | | | | | | | | | | | |
| **Jones (1986)** | | No information on microbiological outcomes. | | | | | | | | | | |
| **5 vs. 10 days First-generation cephalosporin (Cefaclor)** | | | | | | | | | | | | |
| **Hendrickse (1988)** | | No information on microbiological outcomes separated by group. | | | | | | | | | | |
| **5 vs. 10 days Second-generation cephalosporin (Cefuroxime)** | | | | | | | | | | | | |
| **Gooch (1996)** | | | | | | | | | | | | |
| Failure with resistance  Day 11-14 | 26 | | 0 | 0.0 |  | 38 | 1 | 2.6 |  | 0.48 | [0.02; 11.44] | 0.653 |
| **5 vs. 10 days Third-generation cephalosporins (Cefixime, Cefpodoxime)** | | | | | | | | | | | | |
| **Adam (2000)** | | No information on microbiological outcomes. | | | | | | | | | | |
| **Cohen (2000)** | | No information on microbiological outcomes after therapy. | | | | | | | | | | |
| CI: Confidence interval | | | | | | | | | | | | |

| Table S7: Eradication of bacteria in included studies | | | | | | | | | | | | |
| --- | --- | --- | --- | --- | --- | --- | --- | --- | --- | --- | --- | --- |
| **Study**  **Outcome**  **Time point** | **Shorter Therapy** | | | |  | **Longer Therapy** | | |  | **Shorter vs. Longer Therapy** | | |
|  | **Analyzed Participants** | | **Participants with persistent bacteria** | |  | **Analyzed Participants** | **Participants with persistent bacteria** | |  | **Risk ratio** | **[95 %-CI]** | **p-Value** |
|  |  |  | **n** | **%** |  |  | **n** | **%** |  |  |  |  |
| **2 vs. 7 days Penicillin V** | | | | | | | | | | | | |
| **Meistrup-Larsen (1983)** | | No information on microbiological outcomes. | | | | | | | | | | |
| **5 vs. 10 days Penicillin V** | | | | | | | | | | | | |
| **Ingvarsson (1982)** | | | | | | | | | | | | |
| Continued detection of pathogenic bacteria  Day 10 | 66 | | 31 | 47 |  | 68 | 38 | 56 |  | 0.84 | [0.60; 1.17] | 0.305 |
| **3 vs. 10 days Amoxicillin** | | | | | | | | | | | | |
| **Chaput de Saintonge (1982)** | | No information on microbiological outcomes. | | | | | | | | | | |
| **3 vs. 10 days Amoxicillin** | | | | | | | | | | | | |
| **Mandel (1995)** | | No information on microbiological outcomes. | | | | | | | | | | |
| **5 vs. 10 days Amoxicillin-clavulanic acid** | | | | | | | | | | | | |
| **Hoberman (1997)** | | No information on microbiological outcomes. | | | | | | | | | | |
| **Cohen (1998)** | | | | | | | | | | | | |
| Carriage of M. catarrhalis  Day 12-14 | 165 | | 42 | 25.4 |  | 175 | 20 | 11.4 |  | **2.23** | **[1.37; 3.63]** | 0.001 |
| **Hoberman (2016)** | | No information on eradication of bacteria after therapy. | | | | | | | | | | |
| **3 vs. 7 days First-generation cephalosporin (Cefaclor)** | | | | | | | | | | | | |
| **Jones (1986)** | | No information on microbiological outcomes. | | | | | | | | | | |
| **5 vs. 10 days First-generation cephalosporin (Cefaclor)** | | | | | | | | | | | | |
| **Hendrickse (1988)** | | No information on microbiological outcomes separated by group. | | | | | | | | | | |
| **5 vs. 10 days Second-generation cephalosporin (Cefuroxime)** | | | | | | | | | | | | |
| **Gooch (1996)** | | | | | | | | | | | | |
| Unsatisfactory bacteriologic outcome  Day 11-14 | 26 | | 2 | 7.7 |  | 38 | 6 | 15.8 |  | 0.49 | [0.11; 2.23] | 0.354 |
| - *Failure* | *26* | | *2* | *7.7* |  | *38* | *3* | *7.9* |  | *0.97* | *[0.17; 5.43]* | *0.976* |
| - *Failure with resistance* | *26* | | *0* | *0.0* |  | *38* | *1* | *2.6* |  | *0.48* | *[0.02; 11.44]* | *0.653* |
| - *Cure with superinfection* | *26* | | *0* | *0.0* |  | *38* | *2* | *5.3* |  | *0.29* | *[0.01; 5.81]* | *0.419* |
| **5 vs. 10 days Third-generation cephalosporins (Cefixime, Cefpodoxime)** | | | | | | | | | | | | |
| **Adam (2000)** | | No information on microbiological outcomes. | | | | | | | | | | |
| **Cohen (2000)** | | No information on microbiological outcomes after therapy. | | | | | | | | | | |
| CI: Confidence interval | | | | | | | | | | | | |

## **Supplementary methods**

### ***Assessment of clinical efficacy***

This systematic review was conducted as part of the ThemenCheck report T23-04 "Antibiotic therapy: Does a shorter duration of administration lead to comparable treatment results?" [1] for the Institute for Quality and Efficiency in Health Care (IQWiG). It was registered in PROSPERO (CRD42024519113) and was conducted in accordance with the PRISMA guidelines [2]. A protocol to the review was published prospectively [3]. In addition to AOM, the ThemenCheck report also examined community-acquired pneumonia (CAP) in children and adults. Due to differences in participants and treatment, the disease conditions were investigated separately following a combined search. This paper discusses the results for AOM, while the results for CAP will be presented in a separate publication.

Inclusion criteria: Randomised controlled trials (RCTs) without any restrictions in publication date were included. An available full-text publication in German or English was required. If the PICO criteria (population, intervention, control, outcome) were only partially met, the studies were included if at least 80% of the randomised participants met the inclusion criteria or separate analyses were available.

Population: Children (age <18 years) with acute middle ear infection (AOM) without pre-existing conditions predisposing to a complicated course (e.g. genetic disorders or immunodeficiency) were included.

Intervention: The intervention was a shorter course of oral antibiotic therapy for AOM. The antibiotic agent had to be approved and conducted in an outpatient setting.

Comparison: The comparative treatment was a longer oral outpatient antibiotic therapy with the same antibiotic agent in the same dosage as in the intervention group in an outpatient setting.

Outcomes: We analyzed treatment success, recurrence, mortality, adverse events (including hospitalization), health-related quality of life and, additionally, adherence and microbiological outcomes (bacterial eradication and resistance). Generally, studies were excluded for an outcome if the analysis was based on less than 70% of the randomized participants or if the difference between the study arms in the number of evaluable study participants was more than 15%.

Search: The literature search was conducted according to the PRESS criteria (Peer Review of Electronic Search Strategies)[4]. On 6 February 2024, MEDLINE (via Ovid), Embase (via Ovid) and the Cochrane Library (via Wiley) were searched for RCTs. On 26 February 2024, we searched ClinicalTrials.gov, the International Clinical Trials Registry Platform Search Portal, the EU Clinical Trials Register and the Clinical Trials Information System. In addition, the reference lists of systematic reviews, including HTA reports, were screened. A detailed description of the search strategies for the individual databases and registers can be found below or in the ThemenCheck report [1] and ThemenCheck report protocol [3].

Study selection: First, duplicates were automatically identified and manually removed, followed by the title/abstract screening of the identified studies for potential relevance by two independent reviewers (ACB, LG or CS). Studies considered potentially relevant were then analyzed in full text by two independent reviewers (ACB, LG or CS). The reasons for exclusion (on full-text level) were documented. Discrepancies were resolved through discussion or by consulting the third reviewer. Publications excluded during full-text screening are listed with reasons in the ThemenCheck report [1].

Data extraction: Data extraction was performed for all identified relevant studies in standardized tables. We extracted data on study characteristics, population characteristics and outcomes at all available time points. Only outcome data in dichotomous form were included. Relative risks (RR) with 95% confidence intervals (CI) were selected as effect measures. Data extraction was performed by one person and checked by a second person (AB, ACB or CS). Discrepancies were resolved through discussion or by consulting the third person.

Assessment of risk of bias: The assessment of risk of bias was carried out in accordance with the guidelines and manuals of the IQWiG [5]. The overall assessment evaluated the domains randomization sequence generation, allocation concealment, blinding of patient(s) and treating staff, and results-independent reporting, as well as additional aspects (no bias on study level due to unclear participant flow/ early termination/ cointervention/ interim analysis/ differences in baseline characteristics/ different follow-up periods/ carry-over-effects). The outcome-specific assessment included blinding of outcome assessors, implementation of intention-to-treat (ITT) principle, and results-independent reporting, as well as additional aspects (no bias on outcome level due to differences in baseline characteristics/ different follow-up periods/ early termination). The assessment was performed independently by two persons (AB, ACB or CS), with discrepancies resolved through discussion or by consulting the third person.

Non-inferiority: No advantage can be expected from a shorter course of antibiotic therapy for the outcomes of treatment success, recurrence, mortality or disease-related adverse events. However, possible advantages of a shorter treatment duration could include fewer side effects, lower treatment costs, less development of resistance in bacteria and higher adherence of participants. Therefore, a shorter antibiotic therapy might already be beneficial for individuals and society if the treatment effect differs only to an acceptable and clinically irrelevant extend. Based on the recommendations of the European Medicines Agency (EMA), a 10% deviation was classified as clinically acceptable[6]. Thus, non-inferiority margins of the RR with 95% CI were set at 0.9 (positive event) or 1.1 (negative event). For treatment success, the primary time point was based on the EMA guidelines and was set at 1-2 days after the end of therapy or 14-21 days after the start of therapy[6].

Statistical evaluation: The statistical evaluation was based on methods of the IQWiG[5]. Only studies with the same intervention duration (shorter therapy duration) and the same class of antibiotics used were analyzed together and, where possible, pooled in meta-analyses. A pooled result in the form of a meta-analysis was not used if the heterogeneity was too high or if the 95% CI according to Knapp-Hartung was not informative (95% CI wider than confidence intervals of included studies). In addition to the pooled effect of all studies, a pooled effect with 95% CI of studies with low risk of bias was calculated and presented. If exactly two studies with reported results were available, the fixed-effect model was used; if more than two studies were available, the random-effects model was used. Because of fewer than ten studies in each comparison category, no investigation of publication bias with funnel plot was conducted.

The 95% CI of the RR was interpreted in relation to the non-inferiority margin. If the 95%-CI crossed the defined non-inferiority-margin of 10%, no indication of non-inferiority was deducted. However, this does not imply inferiority of the shorter treatment regimen. If non-inferiority could be deducted, certainty of evidence was derived according to the IQWiG methods[5] in three levels depending on numbers and risk of bias of included studies: "evidence of non-inferiority", "indication of non-inferiority", "possible indication of non-inferiority". For the outcome of adverse events, in cases of non-inferiority, possible superiority of a shorter treatment regimen was also examined.

If there were methodological doubts about included studies, sensitivity analyses were performed. The results were summarized systematically in evidence tables and presented with relative and absolute effects. The statistical analysis was performed in R Version 4.4.1 using the packages *meta, readxl* and *tidyverse.*

### ***Further domains***

To examine economic aspects of shorter antibiotic therapy, the MEDLINE, Embase and HTA databases were searched for comparative studies with statements on cost-effectiveness. In addition, reference lists of systematic reviews were examined. The focus was on studies on AOM and CAP. However, only studies on AOM are included in this publication. A detailed description of the search strategy can be found in the ThemenCheck report [1].

Exploratory searches were conducted in the MEDLINE, ETHMED, Philosopher's Index, AWMF guideline database, HealthcareLCA database and Google Scholar databases to evaluate ecological and social aspects of shorter antibiotic regimens. In addition, websites and the included studies from other domains were examined. The search strategies for the individual domains can be found in the ThemenCheck report [1]. The findings were presented narratively, based on thematic frameworks and incorporating interviews with patients and reflective thoughts [7]. The focus was on aspects of shorter antibiotic therapy for AOM and CAP, with priority given to aspects relevant to AOM.

### ***Changes to the protocol***

Details of the methodology used as well as detailed tables and data on included and excluded studies, are provided in the ThemenCheck report protocol[3] and in the ThemenCheck report[1]. Important outcomes such as morbidity or AEs were specified. Generally described methods were adapted to the question of non-inferiority. The non-inferiority margin and time points were determined based on EMA recommendations.

1. Institut für Qualität und Wirtschaftlichkeit im Gesundheitswesen (IQWiG). *Antibiotikatherapie; Führt eine verkürzte Einnahmedauer zu vergleichbaren Behandlungsergebnissen? ThemenCheck-Bericht im Auftrag des IQWiG [online].* 2025 04 September 2025]; Available from: <https://dx.doi.org/10.60584/T23-04>.

2. Page, M.J., et al., *The PRISMA 2020 statement: an updated guideline for reporting systematic reviews.* bmj, 2021. **372**.

3. Institut für Qualität und Wirtschaftlichkeit im Gesundheitswesen (IQWiG). *Antibiotikatherapie; Führt eine verkürzte Einnahmedauer zu vergleichbaren Behandlungsergebnissen?* 2024 06 February 2025]; Available from: <https://www.iqwig.de/download/t23-04_verkuerzte-antibiotikatherapie_themencheck-berichtsprotokoll_version1-0.pdf>.

4. McGowan, J., et al., *PRESS peer review of electronic search strategies: 2015 guideline statement.* Journal of Clinical Epidemiology, 2016. **75**: p. 40-46 DOI: 10.1016/j.jclinepi.2016.01.021.

5. Institut für Qualität und Wirtschaftlichkeit im Gesundheitswesen (IQWiG). *Allgemeine Methoden: Version 7.0*. 2023 30 January 2025]; Available from: <https://www.iqwig.de/methoden/allgemeine-methoden_version-7-0.pdf>.

6. European Medicines Agency (EMA) and Committee for Medicinal Products for Human Use (CHMP). *Addendum to the guideline on the evaluation of medicinal products indicated for treatment of bacterial infections*. 2014 30 January 2025]; Available from: <https://www.google.com/url?sa=t&source=web&rct=j&opi=89978449&url=https://www.ema.europa.eu/en/documents/scientific-guideline/addendum-guideline-evaluation-medicinal-products-indicated-treatment-bacterial-infections_en.pdf&ved=2ahUKEwiwvPuU2--HAxVv2QIHHZTkMrEQFnoECBgQAQ&usg=AOvVaw1SDxAqyc8yo_6W_J0XOdZJ>.

7. Lysdahl KB, M.K., Burns J, Chilcott JB, Brönneke JB, Hofmann B. *Guidance for assessing effectiveness, economic aspects, ethical aspects, socio-cultural aspects and legal aspects in complex technologies*. 2016 04 September 2025]; Available from: <https://validatehta.eu/wp-content/uploads/2022/07/3.-Guidance-for-assessing-effectiveness-economic-aspects-ethical-aspects-socio-cultural-aspects-and-legal-aspects-in-complex-technologies.pdf>.

### **Search strategies**

***Clinical efficacy:***

*Search for systematic reviews:*

1. MEDLINE

Ovid MEDLINE(R) ALL 1946 to October 20, 2023

Filters: Systematic review: Wong – High specificity strategy

Wong SS, Wilczynski NL, Haynes RB. Comparison of top-performing search strategies for detecting clinically sound treatment studies and systematic reviews in MEDLINE and EMBASE. J Med Libr Assoc 2006; 94(4): 451-455.

| # | Searches |
| --- | --- |
| 1 | exp anti-bacterial agents/ |
| 2 | (antibiotic* or anti-biotic* or antibacterial* or anti-bacterial* or antimicrobial* or anti-microbial* or antiinfective* or anti-infective*).ti,ab. |
| 3 | or/1-2 |
| 4 | exp Time Factors/ |
| 5 | exp duration of therapy/ |
| 6 | exp Drug Administration Schedule/ |
| 7 | ((short* or long* or standard* or prolong* or treatment or therap* or regime*) adj3 (term* or course* or duration or length or day*)).ti,ab. |
| 8 | or/4-7 |
| 9 | exp Pneumonia/ |
| 10 | (pneumon* or pleuropneumon* or bronchopneumon*).ti,ab. |
| 11 | or/9-10 |
| 12 | exp Otitis Media/ |
| 13 | (acute adj3 (OM or otitis media or ear)).ti,ab. |
| 14 | or/12-13 |
| 15 | or/11,14 |
| 16 | and/3,8,15 |
| 17 | Cochrane database of systematic reviews.jn. |
| 18 | (search or MEDLINE or systematic review).tw. |
| 19 | meta analysis.pt. |
| 20 | or/17-19 |
| 21 | and/16,20 |
| 22 | 21 and (english or german or multilingual or undetermined).lg. |
| 23 | ..l/ 22 yr=2010-Current |

2. International HTA Database

| # | Searches |
| --- | --- |
| 1 | "anti-infective agents"[mh] |
| 2 | "anti-bacterial agents"[mh] |
| 3 | (antibiotic* or anti-biotic* or antibacterial* or anti-bacterial* or antimicrobial* or anti-microbial* or antiinfective* or anti-infective*)[Title] OR (antibiotic* or anti-biotic* or antibacterial* or anti-bacterial* or antimicrobial* or anti-microbial* or antiinfective* or anti-infective*)[abs] |
| 4 | #3 OR #2 OR #1 |
| 5 | "Pneumonia"[mh] |
| 6 | pneumon*[Title] OR pneumon*[abs] |
| 7 | #6 OR #5 |
| 8 | "Otitis Media"[mh] |
| 9 | "otitis media"[Title] OR "otitis media"[abs] |
| 10 | #9 OR #8 |
| 11 | #10 OR #7 |
| 12 | #11 AND #4 |
| 13 | (*) FROM 2010 TO 2023 |
| 14 | #13 AND #12 |

*Search for Randomized controlled trials*

1. MEDLINE

Ovid MEDLINE(R) ALL 1946 to February 05, 2024

Filter: RCT: Lefebvre – Cochrane Highly Sensitive Search Strategy for identifying randomized trials in MEDLINE: sensitivity- and precision-maximizing version (2008 revision), InterTASC Information Specialists SubGroup (2023 revision)

Lefebvre C, Glanville J, Briscoe S et al. Cochrane Handbook for Systematic Reviews of Interventions; Version 6; Technical Supplement to Chapter 4: Searching for and selecting studies [online]. 2019 [Zugriff: 25.07.2022]. URL: <https://training.cochrane.org/handbook/version-6/chapter-4-tech-suppl>.

Glanville J, Lefebvre C, Manson P et al. ISSG Search Filter Resource; The InterTASC Information Specialists' Sub-Group 2006 updated [online]. 2023 [Zugriff: 05.08.2024]. URL: https://sites.google.com/a/york.ac.uk/issg-search-filters-resource/home.

| # | Searches |
| --- | --- |
| 1 | exp Anti-Bacterial Agents/ |
| 2 | exp Carbapenems/ |
| 3 | exp Cephalosporins/ |
| 4 | exp Fluoroquinolones/ |
| 5 | exp Lincosamides/ |
| 6 | exp Macrolides/ |
| 7 | exp Penicillins/ |
| 8 | exp Tetracyclines/ |
| 9 | antibiotic*.ti,ab. |
| 10 | penicillin*.mp. |
| 11 | amox?cillin*.mp. |
| 12 | ampicillin*.mp. |
| 13 | azit?romycin*.mp. |
| 14 | (cefaclor* or cephaclor*).mp. |
| 15 | (cefalexin* or cephalexin*).mp. |
| 16 | cefdinir*.mp. |
| 17 | cefotaxim*.mp. |
| 18 | cefpodoxim*.mp. |
| 19 | cefprozil*.mp. |
| 20 | ceftizox*.mp. |
| 21 | ceftriaxon*.mp. |
| 22 | cefuroxim*.mp. |
| 23 | clarit?romycin*.mp. |
| 24 | clindamycin*.mp. |
| 25 | doxycyclin*.mp. |
| 26 | ertapenem*.mp. |
| 27 | erythromycin*.mp. |
| 28 | imipenem*.mp. |
| 29 | levofloxacin*.mp. |
| 30 | meropenem*.mp. |
| 31 | moxifloxacin*.mp. |
| 32 | trimet?oprim*.mp. |
| 33 | or/1-32 |
| 34 | exp Drug Administration Schedule/ |
| 35 | ((short* or day or days) adj5 (course or therapy or treatment* or regimen*)).ti,ab. |
| 36 | ((((one or "1" or two or "2" or three or "3" or four or "4" or five or "5" or six or "6" or seven or "7" or eight or "8" or nine or "9" or ten or "10") adj1 (day or days)) or (single adj2 dose)) adj5 (therapy or treatment* or regimen*)).ti,ab. |
| 37 | (((single adj2 dose) or ((one or "1" or two or "2" or three or "3" or four or "4" or five or "5") adj1 (day or days))) and ((six or "6" or seven or "7" or eight or "8" or nine or "9" or ten or "10") adj1 (day or days))).ti,ab. |
| 38 | or/34-37 |
| 39 | exp Pneumonia/ |
| 40 | pneumonia?.ti,ab. |
| 41 | or/39-40 |
| 42 | exp Otitis Media/ |
| 43 | (otitis adj1 media).ti,ab. |
| 44 | or/42-43 |
| 45 | exp randomized controlled trial/ |
| 46 | controlled clinical trial.pt. |
| 47 | (randomized or placebo or randomly).ab. |
| 48 | clinical trials as topic.sh. |
| 49 | trial.ti. |
| 50 | or/45-49 |
| 51 | 50 not (exp animals/ not humans.sh.) |
| 52 | 33 and 38 and (41 or 44) and 51 |
| 53 | (animals/ not humans/) or comment/ or editorial/ or exp review/ or meta analysis/ or consensus/ or exp guideline/ |
| 54 | hi.fs. or case report.mp. |
| 55 | or/53-54 |
| 56 | 52 not 55 |
| 57 | 56 and (english or german or multilingual or undetermined).lg. |

2. Embase

Ovid: Embase 1974 to 2024 February 05

Filter: RCT: Wong – Strategy minimizing difference between sensitivity and specificity

Wong SS, Wilczynski NL, Haynes RB. Comparison of top-performing search strategies for detecting clinically sound treatment studies and systematic reviews in MEDLINE and EMBASE. J Med Libr Assoc 2006; 94(4): 451-455.

| # | Searches |
| --- | --- |
| 1 | antibiotic therapy/ |
| 2 | carbapenem derivative/ |
| 3 | exp cephalosporin derivative/ |
| 4 | exp quinolone derivative/ |
| 5 | lincosamides/ |
| 6 | exp macrolides/ |
| 7 | exp penicillin derivative/ |
| 8 | tetracycline/ |
| 9 | antibiotic*.ti,ab. |
| 10 | penicillin*.mp. |
| 11 | amox?cillin*.mp. |
| 12 | ampicillin*.mp. |
| 13 | azit?romycin*.mp. |
| 14 | (cefaclor* or cephaclor*).mp. |
| 15 | (cefalexin* or cephalexin*).mp. |
| 16 | cefdinir*.mp. |
| 17 | cefotaxim*.mp. |
| 18 | cefpodoxim*.mp. |
| 19 | cefprozil*.mp. |
| 20 | ceftizox*.mp. |
| 21 | ceftriaxon*.mp. |
| 22 | cefuroxim*.mp. |
| 23 | clarit?romycin*.mp. |
| 24 | clindamycin*.mp. |
| 25 | doxycyclin*.mp. |
| 26 | ertapenem*.mp. |
| 27 | erythromycin*.mp. |
| 28 | imipenem*.mp. |
| 29 | levofloxacin*.mp. |
| 30 | meropenem*.mp. |
| 31 | moxifloxacin*.mp. |
| 32 | trimet?oprim*.mp. |
| 33 | or/1-32 |
| 34 | treatment duration/ |
| 35 | ((short* or day or days) adj5 (course or therapy or treatment* or regimen*)).ti,ab. |
| 36 | ((((one or "1" or two or "2" or three or "3" or four or "4" or five or "5" or six or "6" or seven or "7" or eight or "8" or nine or "9" or ten or "10") adj1 (day or days)) or (single adj2 dose)) adj5 (therapy or treatment* or regimen*)).ti,ab. |
| 37 | (((single adj2 dose) or ((one or "1" or two or "2" or three or "3" or four or "4" or five or "5") adj1 (day or days))) and ((six or "6" or seven or "7" or eight or "8" or nine or "9" or ten or "10") adj1 (day or days))).ti,ab. |
| 38 | or/34-37 |
| 39 | exp Pneumonia/ or Streptococcus pneumoniae/ or community acquired pneumonia/ |
| 40 | pneumonia?.ti,ab. |
| 41 | or/39-40 |
| 42 | exp "Otitis Media"/ |
| 43 | (otitis adj1 media).ti,ab. |
| 44 | or/42-43 |
| 45 | (random* or double-blind*).tw. |
| 46 | placebo*.mp. |
| 47 | or/45-46 |
| 48 | 33 and 38 and (41 or 44) and 47 |
| 49 | 48 not medline.cr. |
| 50 | 49 not (exp animal/ not exp human/) |
| 51 | 50 not (Conference Abstract or Conference Review or Editorial).pt. |
| 52 | 51 not ((afrikaans or albanian or arabic or armenian or azerbaijani or basque or belorussian or bosnian or bulgarian or catalan or chinese or croatian or czech or danish or dutch or english or esperanto or estonian or finnish or french or gallegan or georgian or german or greek or hebrew or hindi or hungarian or icelandic or indonesian or irish gaelic or italian or japanese or korean or latvian or lithuanian or macedonian or malay or norwegian or persian or polish or polyglot or portuguese or pushto or romanian or russian or scottish gaelic or serbian or slovak or slovene or spanish or swedish or thai or turkish or ukrainian or urdu or uzbek or vietnamese) not (english or german)).lg. |

3. The Cochrane Library

Wiley: Cochrane Central Register of Controlled Trials: Issue 02 of 12, February 2024

| # | Searches |
| --- | --- |
| #1 | [mh "Anti-Bacterial Agents"] |
| #2 | [mh Carbapenems] |
| #3 | [mh Cephalosporins] |
| #4 | [mh Fluoroquinolones] |
| #5 | [mh Lincosamides] |
| #6 | [mh Macrolides] |
| #7 | [mh Penicillins] |
| #8 | [mh Tetracyclines] |
| #9 | antibiotic*:ti,ab |
| #10 | penicillin*:ti,ab,kw |
| #11 | amox?cillin*:ti,ab,kw |
| #12 | ampicillin*:ti,ab,kw |
| #13 | azit?romycin*:ti,ab,kw |
| #14 | (cefaclor* OR cephaclor*):ti,ab,kw |
| #15 | (cefalexin* OR cephalexin*):ti,ab,kw |
| #16 | cefdinir*:ti,ab,kw |
| #17 | cefotaxim*:ti,ab,kw |
| #18 | cefpodoxim*:ti,ab,kw |
| #19 | cefprozil*:ti,ab,kw |
| #20 | ceftizox*:ti,ab,kw |
| #21 | ceftriaxon*:ti,ab,kw |
| #22 | cefuroxim*:ti,ab,kw |
| #23 | clarit?romycin*:ti,ab,kw |
| #24 | clindamycin*:ti,ab,kw |
| #25 | doxycyclin*:ti,ab,kw |
| #26 | ertapenem*:ti,ab,kw |
| #27 | erythromycin*:ti,ab,kw |
| #28 | imipenem*:ti,ab,kw |
| #29 | levofloxacin*:ti,ab,kw |
| #30 | meropenem*:ti,ab,kw |
| #31 | moxifloxacin*:ti,ab,kw |
| #32 | trimet?oprim*:ti,ab,kw |
| #33 | #1 OR #2 OR #3 OR #4 OR #5 OR #6 OR #7 OR #8 OR #9 OR #10 OR #11 OR #12 OR #13 OR #14 OR #15 OR #16 OR #17 OR #18 OR #19 OR #20 OR #21 OR #22 OR #23 OR #24 OR #25 OR #26 OR #27 OR #28 OR #29 OR #30 OR #31 OR #32 |
| #34 | [mh "Drug Administration Schedule"] |
| #35 | ((short* or day or days) NEAR/5 (course or therapy or treatment* or regimen*)):ti,ab |
| #36 | ((((one or "1" or two or "2" or three or "3" or four or "4" or five or "5" or six or "6" or seven or "7" or eight or "8" or nine or "9" or ten or "10") NEAR/1 (day or days)) or (single NEAR/1 dose)) NEAR/5 (therapy or treatment* or regimen*)):ti,ab |
| #37 | (((single NEAR/2 dose) or ((one or "1" or two or "2" or three or "3" or four or "4" or five or "5") NEAR/1 (day or days))) and ((six or "6" or seven or "7" or eight or "8" or nine or "9" or ten or "10") NEAR/1 (day or days))):ti,ab |
| #38 | #35 OR #36 OR #37 |
| #39 | [mh Pneumonia] |
| #40 | pneumonia?:ti,ab |
| #41 | #39 OR #40 |
| #42 | [mh "Otitis Media"] |
| #43 | (otitis NEAR/1 media):ti,ab |
| #44 | #42 OR #43 |
| #45 | #33 AND #38 AND (#41 OR #44) |
| #46 | #45 not (*clinicaltrial*gov* or *trialsearch*who* or *clinicaltrialsregister*eu* or *anzctr*org*au* or *trialregister*nl* or *irct*ir* or *isrctn* or *controlled*trials*com* or *drks*de*):so |
| #47 | #46 not ((language next (afr or ara or aze or bos or bul or car or cat or chi or cze or dan or dut or es or est or fin or fre or gre or heb or hrv or hun or ice or ira or ita or jpn or ko or kor or lit or nor or peo or per or pol or por or pt or rom or rum or rus or slo or slv or spa or srp or swe or tha or tur or ukr or urd or uzb)) not (language near/2 (en or eng or english or ger or german or mul or unknown))) |
| #48 | #47 in Trials |

*Search in Study Registries*

1. ClinicalTrials.gov

U.S. National Institutes of Health (Basic Search), URL: <http://www.clinicaltrials.gov>

| Search |
| --- |
| (pneumonia OR pneumoniae OR otitis OR "lung infection") [Condition/disease] ("antibiotic treatment" OR "antibiotic therapy" OR penicillins OR amoxycillin OR amoxicillin OR BRL-2333 OR ampicillin OR azithromycin OR CP-62993 OR cefaclor OR cephaclor OR S-6472 OR cefalexin OR cephalexin OR cefdinir OR CI-983 OR FK-482 OR PD-134393 OR cefotaxime OR HR-756 OR Ru-24756 OR cefpodoxime OR R-3746 OR RU-51746 OR cefprozil OR BMY-28100 OR ceftizoxime OR FK-749 OR FR-13749 OR ceftriaxone OR Ro-13-9904 OR cefuroxime OR clarithromycin OR A-56268 OR TE-031 OR clindamycin OR doxycycline OR BMY-28689 OR BU-3839T OR ertapenem OR erythromycin OR imipenem OR MK-0787 OR levofloxacin OR meropenem OR SM-7338 OR moxifloxacin OR BAY-128039 OR trimethoprim) AND (short OR reduction OR day OR course OR (single AND dose)) [Intervention/treatment] Filter Study Phase: Phase 2, Phase 3, Phase 4, Not applicable |

2. International Clinical Trials Registry Platform Search Portal

World Health Organization (Standard Search), URL: [https://trialsearch.who.int](https://trialsearch.who.int/)

| Suchstrategie |
| --- |
| (pneumonia* OR otitis* OR "lung infection") AND (antibiotic* OR antimicrobial* OR penicillin* OR amoxycillin* OR amoxicillin* OR BRL-2333 OR BRL2333 OR BRL 2333 OR ampicillin* OR azithromycin* OR CP-62993 OR CP62993 OR CP 62993 OR cefaclor* OR cephaclor* OR S-6472 OR S6472 OR S 6472 OR cefalexin* OR cephalexin* OR cefdinir* OR CI-983 OR CI983 OR CI 983 OR FK-482 OR FK482 OR FK 482 OR PD-134393 OR PD134393 OR PD 134393 OR cefotaxim* OR HR-756 OR HR756 OR HR 756 OR Ru-24756 OR Ru24756 OR Ru 24756 OR cefpodoxim* OR R-3746 OR R3746 OR R 3746 OR RU-51746 OR RU51746 OR RU 51746 OR cefprozil* OR BMY-28100 OR BMY28100 OR BMY 28100 OR ceftizox* OR FK-749 OR FK749 OR FK 749 OR FR-13749 OR FR13749 OR FR 13749 OR ceftriaxon* OR Ro-13-9904 OR Ro139904 OR Ro 13 9904 OR cefuroxim* OR clarithromycin* OR A-56268 OR A56268 OR A 56268 OR TE-031 OR TE031 OR TE 031 OR clindamycin* OR doxycyclin* OR BMY-28689 OR BMY28689 OR BMY 28689 OR BU-3839T OR BU3839T OR BU 3839T OR ertapenem* OR erythromycin* OR imipenem* OR MK-0787 OR MK0787 OR MK 0787 OR levofloxacin* OR meropenem* OR SM-7338 OR SM7338 OR SM 7338 OR moxifloxacin* OR BAY-128039 OR BAY128039 OR BAY 128039 OR trimethoprim*) AND (short* OR reduc* OR day* OR course OR (single AND dose)) |

3. EU Clinical Trials Register

European Medicines Agency (Basic Search), URL: <https://www.clinicaltrialsregister.eu/ctr-search/search>

| Search |
| --- |
| (pneumonia* OR otitis* OR "lung infection") AND (antibiotic* OR antimicrobial* OR penicillin* OR amoxycillin* OR amoxicillin* OR BRL-2333 OR BRL2333 OR (BRL 2333) OR ampicillin* OR azithromycin* OR CP-62993 OR CP62993 OR (CP 62993) OR cefaclor* OR cephaclor* OR S-6472 OR S6472 OR (S 6472) OR cefalexin* OR cephalexin* OR cefdinir* OR CI-983 OR CI983 OR (CI 983) OR FK-482 OR FK482 OR (FK 482) OR PD-134393 OR PD134393 OR (PD 134393) OR cefotaxim* OR HR-756 OR HR756 OR (HR 756) OR Ru-24756 OR Ru24756 OR (Ru 24756) OR cefpodoxim* OR R-3746 OR R3746 OR (R 3746) OR RU-51746 OR RU51746 OR (RU 51746) OR cefprozil* OR BMY-28100 OR BMY28100 OR (BMY 28100) OR ceftizox* OR FK-749 OR FK749 OR (FK 749) OR FR-13749 OR FR13749 OR (FR 13749) OR ceftriaxon* OR Ro-13-9904 OR Ro139904 OR (Ro 13 9904) OR cefuroxim* OR clarithromycin* OR A-56268 OR A56268 OR (A 56268) OR TE-031 OR TE031 OR (TE 031) OR clindamycin* OR doxycyclin* OR BMY-28689 OR BMY28689 OR (BMY 28689) OR BU-3839T OR BU3839T OR (BU 3839T) OR ertapenem* OR erythromycin* OR imipenem* OR MK-0787 OR MK0787 OR (MK 0787) OR levofloxacin* OR meropenem* OR SM-7338 OR SM7338 OR (SM 7338) OR moxifloxacin* OR BAY-128039 OR BAY128039 OR (BAY 128039) OR trimethoprim*) AND (short* OR reduc* OR day* OR course OR (single AND dose)) |

4. Clinical Trials Information System

European Medicines Agency (Basic Search), URL: <https://euclinicaltrials.eu/search-for-clinical-trials/?lang=en>

| Search |
| --- |
| antibiotic, antibiotics, antimicrobial, antimicrobials, penicillin, penicillins, amoxycillin, amoxicillin, BRL-2333, BRL2333, BRL 2333, ampicillin, azithromycin, CP-62993, CP62993, CP 62993, cefaclor, cephaclor, S-6472, S6472, S 6472, cefalexin, cephalexin, cefdinir, CI-983, CI983, CI 983, FK-482, FK482, FK 482, PD-134393, PD134393, PD 134393, cefotaxime, HR-756, HR756, HR 756, Ru-24756, Ru24756, Ru 24756, cefpodoxime, R-3746, R3746, R 3746, RU-51746, RU51746, RU 51746, cefprozil, BMY-28100, BMY28100, BMY 28100, ceftizoxime, FK-749, FK749, FK 749, FR-13749, FR13749, FR 13749, ceftriaxon, ceftriaxone, Ro-13-9904, Ro139904, Ro 13 9904, cefuroxime, clarithromycin, A-56268, A56268, A 56268, TE-031, TE031, TE 031, clindamycin, doxycycline, BMY-28689, BMY28689, BMY 28689, BU-3839T, BU3839T, BU 3839T, ertapenem, erythromycin, imipenem, MK-0787, MK0787, MK 0787, levofloxacin, meropenem, SM-7338, SM7338, SM 7338, moxifloxacin, BAY-128039, BAY128039, BAY 128039, trimethoprim |

***Economic aspects:***

1. MEDLINE

Ovid MEDLINE(R) ALL 1946 to February 13, 2024

Filter: Health economics study: Glanville, Fleetwood

Glanville J, Fleetwood K, Yellowlees A et al. Development and Testing of Search Filters to Identify Economic Evaluations in MEDLINE and EMBASE [online]. 2009 [Zugriff: 25.07.2022]. URL: <https://www.cadth.ca/media/pdf/H0490_Search_Filters_for_Economic_Evaluations_mg_e.pdf>.

| # | Searches |
| --- | --- |
| 1 | exp Anti-Bacterial Agents/ |
| 2 | exp Carbapenems/ |
| 3 | exp Cephalosporins/ |
| 4 | exp Fluoroquinolones/ |
| 5 | exp Lincosamides/ |
| 6 | exp Macrolides/ |
| 7 | exp Penicillins/ |
| 8 | exp Tetracyclines/ |
| 9 | antibiotic*.ti,ab. |
| 10 | penicillin*.mp. |
| 11 | amox?cillin*.mp. |
| 12 | ampicillin*.mp. |
| 13 | azit?romycin*.mp. |
| 14 | (cefaclor* or cephaclor*).mp. |
| 15 | (cefalexin* or cephalexin*).mp. |
| 16 | cefdinir*.mp. |
| 17 | cefotaxim*.mp. |
| 18 | cefpodoxim*.mp. |
| 19 | cefprozil*.mp. |
| 20 | ceftizox*.mp. |
| 21 | ceftriaxon*.mp. |
| 22 | cefuroxim*.mp. |
| 23 | clarit?romycin*.mp. |
| 24 | clindamycin*.mp. |
| 25 | doxycyclin*.mp. |
| 26 | ertapenem*.mp. |
| 27 | erythromycin*.mp. |
| 28 | imipenem*.mp. |
| 29 | levofloxacin*.mp. |
| 30 | meropenem*.mp. |
| 31 | moxifloxacin*.mp. |
| 32 | trimet?oprim*.mp. |
| 33 | or/1-32 |
| 34 | exp Drug Administration Schedule/ |
| 35 | ((short* or day or days) adj5 (course or therapy or treatment* or regimen*)).ti,ab. |
| 36 | ((((one or "1" or two or "2" or three or "3" or four or "4" or five or "5" or six or "6" or seven or "7" or eight or "8" or nine or "9" or ten or "10") adj1 (day or days)) or (single adj2 dose)) adj5 (therapy or treatment* or regimen*)).ti,ab. |
| 37 | (((single adj2 dose) or ((one or "1" or two or "2" or three or "3" or four or "4" or five or "5") adj1 (day or days))) and ((six or "6" or seven or "7" or eight or "8" or nine or "9" or ten or "10") adj1 (day or days))).ti,ab. |
| 38 | or/34-37 |
| 39 | exp Pneumonia/ |
| 40 | pneumonia?.ti,ab. |
| 41 | or/39-40 |
| 42 | exp Otitis Media/ |
| 43 | (otitis adj1 media).ti,ab. |
| 44 | or/42-43 |
| 45 | (economic$ or cost$).ti. |
| 46 | cost benefit analysis/ |
| 47 | treatment outcome/ and ec.fs. |
| 48 | or/45-47 |
| 49 | 48 not ((animals/ not humans/) or letter.pt.) |
| 50 | 33 and 38 and (41 or 44) and 49 |
| 51 | 50 not (comment or editorial).pt. |
| 52 | 51 and (english or german or multilingual or undetermined).lg. |

2. Embase

Ovid: Embase 1974 to 2024 February 13

Filter: Health economics study: Glanville, Fleetwood

Glanville J, Fleetwood K, Yellowlees A et al. Development and Testing of Search Filters to Identify Economic Evaluations in MEDLINE and EMBASE [online]. 2009 [Zugriff: 25.07.2022]. URL: <https://www.cadth.ca/media/pdf/H0490_Search_Filters_for_Economic_Evaluations_mg_e.pdf>.

| # | Searches |
| --- | --- |
| 1 | antibiotic therapy/ |
| 2 | carbapenem derivative/ |
| 3 | exp cephalosporin derivative/ |
| 4 | exp quinolone derivative/ |
| 5 | lincosamides/ |
| 6 | exp macrolides/ |
| 7 | exp penicillin derivative/ |
| 8 | tetracycline/ |
| 9 | antibiotic*.ti,ab. |
| 10 | penicillin*.mp. |
| 11 | amox?cillin*.mp. |
| 12 | ampicillin*.mp. |
| 13 | azit?romycin*.mp. |
| 14 | (cefaclor* or cephaclor*).mp. |
| 15 | (cefalexin* or cephalexin*).mp. |
| 16 | cefdinir*.mp. |
| 17 | cefotaxim*.mp. |
| 18 | cefpodoxim*.mp. |
| 19 | cefprozil*.mp. |
| 20 | ceftizox*.mp. |
| 21 | ceftriaxon*.mp. |
| 22 | cefuroxim*.mp. |
| 23 | clarit?romycin*.mp. |
| 24 | clindamycin*.mp. |
| 25 | doxycyclin*.mp. |
| 26 | ertapenem*.mp. |
| 27 | erythromycin*.mp. |
| 28 | imipenem*.mp. |
| 29 | levofloxacin*.mp. |
| 30 | meropenem*.mp. |
| 31 | moxifloxacin*.mp. |
| 32 | trimet?oprim*.mp. |
| 33 | or/1-32 |
| 34 | treatment duration/ |
| 35 | ((short* or day or days) adj5 (course or therapy or treatment* or regimen*)).ti,ab. |
| 36 | ((((one or "1" or two or "2" or three or "3" or four or "4" or five or "5" or six or "6" or seven or "7" or eight or "8" or nine or "9" or ten or "10") adj1 (day or days)) or (single adj2 dose)) adj5 (therapy or treatment* or regimen*)).ti,ab. |
| 37 | (((single adj2 dose) or ((one or "1" or two or "2" or three or "3" or four or "4" or five or "5") adj1 (day or days))) and ((six or "6" or seven or "7" or eight or "8" or nine or "9" or ten or "10") adj1 (day or days))).ti,ab. |
| 38 | or/34-37 |
| 39 | exp Pneumonia/ or Streptococcus pneumoniae/ or community acquired pneumonia/ |
| 40 | pneumonia?.ti,ab. |
| 41 | or/39-40 |
| 42 | exp "Otitis Media"/ |
| 43 | (otitis adj1 media).ti,ab. |
| 44 | or/42-43 |
| 45 | (Cost adj effectiveness).ab. |
| 46 | (Cost adj effectiveness).ti. |
| 47 | (Life adj years).ab. |
| 48 | (Life adj year).ab. |
| 49 | Qaly.ab. |
| 50 | (Cost or costs).ab. and Controlled Study/ |
| 51 | (Cost and costs).ab. |
| 52 | or/45-51 |
| 53 | 33 and 38 and (41 or 44) and 52 |
| 54 | 53 not medline.cr. |
| 55 | 54 not (exp animal/ not exp human/) |
| 56 | 55 not (Conference Abstract or Conference Review or Editorial).pt. |
| 57 | 56 and (english or german).lg. |

3. International HTA Database

| # | Searches |
| --- | --- |
| #1 | "Anti-Bacterial Agents"[mhe] |
| #2 | Carbapenems[mhe] |
| #3 | Cephalosporins[mhe] |
| #4 | Fluoroquinolones[mhe] |
| #5 | Lincosamides[mhe] |
| #6 | Macrolides[mhe] |
| #7 | Penicillins[mhe] |
| #8 | Tetracyclines[mhe] |
| #9 | (antibiotic* OR penicillin* OR amoxycillin* OR amoxicillin* OR ampicillin* OR azithromycin* OR cefaclor* OR cephaclor* OR cefalexin* OR cephalexin* OR cefdinir* OR cefotaxim* OR cefpodoxim* OR cefprozil* OR ceftizox* OR ceftriaxon* OR cefuroxim* OR clarithromycin* OR clindamycin* OR doxycyclin* OR ertapenem* OR erythromycin* OR imipenem* OR levofloxacin* OR meropenem* OR moxifloxacin* OR trimethoprim*)[title] OR (antibiotic* OR penicillin* OR amoxycillin* OR amoxicillin* OR ampicillin* OR azithromycin* OR cefaclor* OR cephaclor* OR cefalexin* OR cephalexin* OR cefdinir* OR cefotaxim* OR cefpodoxim* OR cefprozil* OR ceftizox* OR ceftriaxon* OR cefuroxim* OR clarithromycin* OR clindamycin* OR doxycyclin* OR ertapenem* OR erythromycin* OR imipenem* OR levofloxacin* OR meropenem* OR moxifloxacin* OR trimethoprim*)[abs] |
| #10 | #9 OR #8 OR #7 OR #6 OR #5 OR #4 OR #3 OR #2 OR #1 |
| #11 | Pneumonia[mhe] |
| #12 | pneumonia*[title] OR pneumonia*[abs] |
| #13 | #12 OR #11 |
| #14 | "Otitis Media"[mhe] |
| #15 | "otitis media"[title] OR "otitis media"[abs] |
| #16 | #15 OR #14 |
| #17 | #16 OR #13 |
| #18 | #17 AND #10 |

List of screened systematic reviews

1. Ben-Shimol S, Levy-Litan V, Falup-Pecurariu O, Greenberg D. Evidence for short duration of antibiotic treatment for non-severe community acquired pneumonia (CAP) in children - are we there yet? A systematic review of randomised controlled trials. Pneumonia 2014; 4: 16-23. https://dx.doi.org/10.15172/pneu.2014.4/432.
2. Chee E, Huang K, Haggie S, Britton PN. Systematic review of clinical practice guidelines on the management of community acquired pneumonia in children. Paediatr Respir Rev 2022; 42: 59-68. https://dx.doi.org/10.1016/j.prrv.2022.01.006.
3. Dawson-Hahn EE, Mickan S, Onakpoya I et al. Short-course versus long-course oral antibiotic treatment for infections treated in outpatient settings: a review of systematic reviews. Fam Pract 2017; 34(5): 511-519. https://dx.doi.org/10.1093/fampra/cmx037.
4. Furlan L, Erba L, Trombetta L et al. Short- vs long-course antibiotic therapy for pneumonia: a comparison of systematic reviews and guidelines for the SIMI Choosing Wisely Campaign. Intern Emerg Med 2019; 14(3): 377-394. https://dx.doi.org/10.1007/s11739-018-1955-2.
5. Furukawa Y, Luo Y, Funada S et al. Optimal duration of antibiotic treatment for community-acquired pneumonia in adults: a systematic review and duration-effect meta-analysis. BMJ Open 2023; 13(3): e061023. https://dx.doi.org/10.1136/bmjopen-2022-061023.
6. Gao Y, Liu M, Yang K et al. Shorter Versus Longer-term Antibiotic Treatments for Community-Acquired Pneumonia in Children: A Meta-analysis. Pediatrics 2023; 151(6): e2022060097. https://dx.doi.org/10.1542/peds.2022-060097.
7. Gulani A, Sachdev HP, Qazi SA. Efficacy of short course (<4 days) of antibiotics for treatment of acute otitis media in children: a systematic review of randomized controlled trials. Indian Pediatr 2010; 47(1): 74-87. https://dx.doi.org/10.1007/s13312-010-0010-9.
8. Hanretty AM, Gallagher JC. Shortened Courses of Antibiotics for Bacterial Infections: A Systematic Review of Randomized Controlled Trials. Pharmacotherapy 2018; 38(6): 674-687. https://dx.doi.org/10.1002/phar.2118.
9. I RM, I PC, S AC et al. Shorter versus longer duration of Amoxicillin-based treatment for pediatric patients with community-acquired pneumonia: a systematic review and meta-analysis. Eur J Pediatr 2022; 181(11): 3795-3804. https://dx.doi.org/10.1007/s00431-022-04603-8.
10. Kim MS, Kim JH, Ryu S et al. Comparative efficacy and optimal duration of first-line antibiotic regimens for acute otitis media in children and adolescents: a systematic review and network meta-analysis of 89 randomized clinical trials. World J Pediatr 2023. https://dx.doi.org/10.1007/s12519-023-00716-8.
11. Kozyrskyj A, Klassen TP, Moffatt M, Harvey K. Short-course antibiotics for acute otitis media. Cochrane Database Syst Rev 2010; (9): CD001095. https://dx.doi.org/10.1002/14651858.CD001095.pub2.
12. Kuitunen I, Jaaskelainen J, Korppi M, Renko M. Antibiotic Treatment Duration for Community-Acquired Pneumonia in Outpatient Children in High-Income Countries-A Systematic Review and Meta-Analysis. Clin Infect Dis 2023; 76(3): e1123-e1128. https://dx.doi.org/10.1093/cid/ciac374.
13. Lan SH, Lai CC, Chang SP et al. Five-day antibiotic treatment for community-acquired bacterial pneumonia: A systematic review and meta-analysis of randomized controlled trials. Journal of Global Antimicrobial Resistance 2020; 23: 94-99. https://dx.doi.org/10.1016/j.jgar.2020.08.005.
14. Lassi ZS, Das JK, Haider SW et al. Systematic review on antibiotic therapy for pneumonia in children between 2 and 59 months of age. Arch Dis Child 2014; 99(7): 687-693. https://dx.doi.org/10.1136/archdischild-2013-304023.
15. Li Q, Zhou Q, Florez ID et al. Short-Course vs Long-Course Antibiotic Therapy for Children With Nonsevere Community-Acquired Pneumonia: A Systematic Review and Meta-analysis. JAMA Pediatrics 2022; 176(12): 1199-1207. https://dx.doi.org/10.1001/jamapediatrics.2022.4123.
16. Lopez-Alcalde J, Rodriguez-Barrientos R, Redondo-Sanchez J et al. Short-course versus long-course therapy of the same antibiotic for community-acquired pneumonia in adolescent and adult outpatients. Cochrane Database Syst Rev 2018; 9: CD009070. https://dx.doi.org/10.1002/14651858.CD009070.pub2.
17. Moller Gundersen K, Nygaard Jensen J, Bjerrum L, Hansen MP. Short-course vs long-course antibiotic treatment for community-acquired pneumonia: A literature review. Basic Clin Pharmacol Toxicol 2019; 124(5): 550-559. https://dx.doi.org/10.1111/bcpt.13205.
18. National Institute for H, Care E. Pneumonia (community-acquired): antimicrobial prescribing [online]. 2019 [Zugriff: 23.10.2023]. URL: https://www.nice.org.uk/guidance/ng138.
19. National Institute for H, Care E. Pneumonia in adults: diagnosis and management [online]. 2022 [Zugriff: 23.10.2023]. URL: https://www.nice.org.uk/guidance/cg191.
20. National Institute for H, Care E. Otitis media (acute): antimicrobial prescribing [online]. 2022 [Zugriff: 23.10.2023]. URL: https://www.nice.org.uk/guidance/ng91.
21. Suzuki HG, Dewez JE, Nijman RG, Yeung S. Clinical practice guidelines for acute otitis media in children: a systematic review and appraisal of European national guidelines. BMJ Open 2020; 10(5): e035343. https://dx.doi.org/10.1136/bmjopen-2019-035343.
22. Tansarli GS, Mylonakis E. Systematic Review and Meta-analysis of the Efficacy of Short-Course Antibiotic Treatments for Community-Acquired Pneumonia in Adults. Antimicrob Agents Chemother 2018; 62(9). https://dx.doi.org/10.1128/aac.00635-18.
23. Team DTB. Antibiotic course length for pneumonia in young children. Drug Ther Bull 2023; 61(8): 117. <https://dx.doi.org/10.1136/dtb.2023.000038>.

Liste of excluded studies (on full text level)

Population not relevant (n=3)

1. Anonymous. Erratum: Effectiveness of discontinuing antibiotic treatment after three days versus eight days in mild to moderate-severe community acquired pneumonia: Randomised, double blind study (British Medical Journal (June 10, 2006) 332 (1355-1358)). Br Med J 2006; 333(7570): 690.
2. Fekete T. In moderately severe CAP stable after 3 d of β-lactam, stopping therapy was noninferior to 5 additional d. Ann Intern Med 2021; 174(8): Jc87. https://dx.doi.org/10.7326/acpj202108170-087.
3. Thomsen J, Sederberg-Olsen J, Balle V, Hartzen S. Antibiotic treatment of children with secretory otitis media. Amoxicillin-clavulanate is superior to penicillin V in a double-blind randomized study. Archives of Otolaryngology -- Head & Neck Surgery 1997; 123(7): 695-699. https://dx.doi.org/10.1001/archotol.1997.01900070039006.

Intervention not relevant (n=5)

1. File TM, Jr., Mandell LA, Tillotson G et al. Gemifloxacin once daily for 5 days versus 7 days for the treatment of community-acquired pneumonia: a randomized, multicentre, double-blind study. J Antimicrob Chemother 2007; 60(1): 112-120. https://dx.doi.org/10.1093/jac/dkm119.
2. Kafetzis DA, Astra H, Mitropoulos L. Five-day versus ten-day treatment of acute otitis media with cefprozil. European Journal of Clinical Microbiology & Infectious Diseases 1997; 16(4): 283-286. https://dx.doi.org/10.1007/bf01695632.
3. Roos K, Larsson P. Efficacy of ceftibuten in 5 versus 10 days treatment of recurrent acute otitis media in children. Int J Pediatr Otorhinolaryngol 2000; 55(2): 109-115. https://dx.doi.org/10.1016/s0165-5876(00)00383-9.
4. Simon MW. Five- vs 10-day treatment of acute otitis media with ceftibuten in infants and children. Adv Ther 1997; 14(6): 312‐317.
5. Tellier G, Chang JR, Asche CV et al. Comparison of hospitalization rates in patients with community-acquired pneumonia treated with telithromycin for 5 or 7 days or clarithromycin for 10 days. Curr Med Res Opin 2004; 20(5): 739-747. https://dx.doi.org/10.1185/030079904125003610.

Comparison not relevant (n=27)

1. Adam D. Five-day therapy with cefpodoxime versus ten-day treatment with cefaclor in infants with acute otitis media. Infection 1995; 23(6): 398-400. https://dx.doi.org/10.1007/bf01713583.
2. Arguedas A, Loaiza C, Perez A et al. A pilot study of single-dose azithromycin versus three-day azithromycin or single-dose ceftriaxone for uncomplicated acute otitis media in children. Curr Ther Res Clin Exp 2003; 64: 16-29. https://dx.doi.org/10.1016/j.curtheres.2003.09.005.
3. Arguedas A, Loaiza C, Rodriguez F et al. Comparative trial of 3 days of azithromycin versus 10 days of clarithromycin in the treatment of children with acute otitis media with effusion. J Chemother 1997; 9(1): 44-50. https://dx.doi.org/10.1179/joc.1997.9.1.44.
4. Arguedas A, Loaiza C, Soley C. Single dose azithromycin for the treatment of uncomplicated otitis media. Pediatr Infect Dis J 2004; 23(2): S108-S114. https://dx.doi.org/10.1097/01.inf.0000112524.71839.c6.
5. Bain J, Murphy E, Ross F. Acute otitis media: clinical course among children who received a short course of high dose antibiotic. British Medical Journal Clinical Research Ed 1985; 291(6504): 1243-1246. https://dx.doi.org/10.1136/bmj.291.6504.1243.
6. Block SL, Kratzer J, Nemeth MA, Tack KJ. Five-day cefdinir course vs. ten-day cefprozil course for treatment of acute otitis media. Pediatr Infect Dis J 2000; 19(12 Suppl): S147-152. https://dx.doi.org/10.1097/00006454-200012001-00003.
7. Daniel R. Simplified treatment of acute lower respiratory tract infection with azithromycin: a comparison with erythromycin and amoxycillin. European Azithromycin Study Group. J Int Med Res 1991; 19(5): 373-383. https://dx.doi.org/10.1177/030006059101900503.
8. Drehobl MA, De Salvo MC, Lewis DE, Breen JD. Single-dose azithromycin microspheres vs clarithromycin extended release for the treatment of mild-to-moderate community-acquired pneumonia in adults. Chest 2005; 128(4): 2230-2237. https://dx.doi.org/10.1378/chest.128.4.2230.
9. Dunbar LM, Khashab MM, Kahn JB et al. Efficacy of 750-mg, 5-day levofloxacin in the treatment of community-acquired pneumonia caused by atypical pathogens. Curr Med Res Opin 2004; 20(4): 555-563. https://dx.doi.org/10.1185/030079904125003304.
10. Dunbar LM, Wunderink RG, Habib MP et al. High-dose, short-course levofloxacin for community-acquired pneumonia: a new treatment paradigm. Clin Infect Dis 2003; 37(6): 752-760. https://dx.doi.org/10.1086/377539.
11. Ficnar B, Huzjak N, Oreskovic K et al. Azithromycin: 3-day versus 5-day course in the treatment of respiratory tract infections in children. Croatian Azithromycin Study Group. J Chemother 1997; 9(1): 38-43. https://dx.doi.org/10.1179/joc.1997.9.1.38.
12. File TM, Jr., Milkovich G, Tennenberg AM et al. Clinical implications of 750 mg, 5-day levofloxacin for the treatment of community-acquired pneumonia. Curr Med Res Opin 2004; 20(9): 1473-1481. https://dx.doi.org/10.1185/030079904x2556.
13. McLinn S. A multicenter, double blind comparison of azithromycin and amoxicillin/ clavulanate for the treatment of acute otitis media in children. Pediatr Infect Dis J 1996; 15(9 Suppl): S20-23. https://dx.doi.org/10.1097/00006454-199609009-00004.
14. Morris PS, Gadil G, McCallum GB et al. Single-dose azithromycin versus seven days of amoxycillin in the treatment of acute otitis media in Aboriginal children (AATAAC): a double blind, randomised controlled trial. Med J Aust 2010; 192(1): 24-29. https://dx.doi.org/10.5694/j.1326-5377.2010.tb03396.x.
15. Muller O. An open comparative study of azithromycin and roxithromycin in the treatment of acute upper respiratory tract infections. J Antimicrob Chemother 1996; 37 Suppl C: 83-92. https://dx.doi.org/10.1093/jac/37.suppl_c.83.
16. O'Doherty B, Muller O. Randomized, multicentre study of the efficacy and tolerance of azithromycin versus clarithromycin in the treatment of adults with mild to moderate community-acquired pneumonia. Azithromycin Study Group. Eur J Clin Microbiol Infect Dis 1998; 17(12): 828-833. https://dx.doi.org/10.1007/s100960050201.
17. Ogale S, Oke V, Bowalekar S et al. Comparative evaluation of the efficacy and safety of azithromycin and roxithromycin in children suffering from otitis media. Indian Pract 2000; 53(10): 651‐657.
18. Orienti S, Colombo F, Gervasini A et al. Ceftriaxone versus cefotaxime in the therapy of pulmonary infections. Current Therapeutic Research - Clinical and Experimental 1989; 45(5): 733-737.
19. Puczynski MS, Stankiewicz JA, O'Keefe JP. Single dose amoxicillin treatment of acute otitis media. Laryngoscope 1987; 97(1): 16-18. https://dx.doi.org/10.1288/00005537-198701000-00006.
20. Rizzato G, Montemurro L, Fraioli P et al. Efficacy of a three day course of azithromycin in moderately severe community-acquired pneumonia. Eur Respir J 1995; 8(3): 398-402. https://dx.doi.org/10.1183/09031936.95.08030398.
21. Ronchetti R, Blasi F, Grossi E, Pecori A. The role of azithromycin in treating children with community-acquired pneumonia. Current therapeutic research clinical and experimental 1994; 55(8): 965‐970. https://dx.doi.org/10.1016/s0011-393x(05)80588-0.
22. Schonwald S, Barsic B, Klinar I, Gunjaca M. Three-day azithromycin compared with ten-day roxithromycin treatment of atypical pneumonia. Scand J Infect Dis 1994; 26(6): 706-710. https://dx.doi.org/10.3109/00365549409008639.
23. Schonwald S, Gunjaca M, Kolacny-Babic L et al. Comparison of azithromycin and erythromycin in the treatment of atypical pneumonias. J Antimicrob Chemother 1990; 25 Suppl A: 123-126. https://dx.doi.org/10.1093/jac/25.suppl_a.123.
24. Schonwald S, Skerk V, Petricevic I et al. Comparison of three-day and five-day courses of azithromycin in the treatment of atypical pneumonia. Eur J Clin Microbiol Infect Dis 1991; 10(10): 877-880. https://dx.doi.org/10.1007/bf01975847.
25. Schrag SJ, Pena C, Fernandez J et al. Effect of short-course, high-dose amoxicillin therapy on resistant pneumococcal carriage: a randomized trial. JAMA 2001; 286(1): 49-56. https://dx.doi.org/10.1001/jama.286.1.49.
26. Shorr AF, Zadeikis N, Xiang JX et al. A multicenter, randomized, double-blind, retrospective comparison of 5- and 10-day regimens of levofloxacin in a subgroup of patients aged > or =65 years with community-acquired pneumonia. Clin Ther 2005; 27(8): 1251-1259. https://dx.doi.org/10.1016/s0149-2918(05)80214-0.
27. Stenstrom C, Lundgren K, Ingvarsson L, Bertilson SO. Amoxycillin/clavulanate versus amoxycillin in recurrent otitis media and therapeutic failure in children. Acta Otolaryngol 1991; 111(1): 120-129. https://dx.doi.org/10.3109/00016489109137362.

Outcomes not relevant (n=0)

Study type not relevant (n=30)

1. Agarwal A, Gao Y, Colunga Lozano LE et al. Shorter versus longer durations of antibiotic treatment for patients with community-acquired pneumonia: a protocol for a systematic review and meta-analysis. BMJ Open 2022; 12(6): e062428. https://dx.doi.org/10.1136/bmjopen-2022-062428.
2. Anonymous. Pneumonia can be treated with 3-5 days of ABX. J Natl Med Assoc 2008; 100(1): 151. https://dx.doi.org/10.1016/s0027-9684(15)31189-5.
3. Anonymous. Evidence for shorter antibiotic courses for pneumonia in children. Drug Ther Bull 2022; 60(11): 164. <https://dx.doi.org/10.1136/dtb.2022.000053>.
4. Barry, H. C. (2021). "Five Days of Antibiotic Therapy Is Comparable with 10 Days in Children with Community-Acquired Pneumonia." American Family Physician 104(5): Online.
5. Blanc A, Cameron J, Yeats A. A narrative review of short versus long-course antibiotic treatment for community-acquired pneumonia in the outpatient pediatric setting. Journal de Pharmacie Clinique 2022; 41(2): 57-65. https://dx.doi.org/10.1684/jpc.2022.0484.
6. Dawson-Hahn EE, Mickan S, Onakpoya I et al. Short-course versus long-course oral antibiotic treatment for infections treated in outpatient settings: A review of systematic reviews. Fam Pract 2017; 34(5): 511-519. https://dx.doi.org/10.1093/fampra/cmx037.
7. Dimopoulos G, Matthaiou DK, Karageorgopoulos DE et al. Short- versus long-course antibacterial therapy for community-acquired pneumonia: A meta-analysis. Drugs 2008; 68(13): 1841-1854. https://dx.doi.org/10.2165/00003495-200868130-00004.
8. Forsch RT. Short-course antibiotics for acute otitis media. J Fam Pract 1998; 47(3): 173‐174.
9. Furukawa Y, Luo Y, Funada S et al. Optimal duration of antibiotic treatment for community-acquired pneumonia in adults: A systematic review and duration-effect meta-analysis. BMJ Open 2023; 13(3): e061023. https://dx.doi.org/10.1136/bmjopen-2022-061023.
10. Gao Y, Liu M, Yang K et al. Shorter Versus Longer-term Antibiotic Treatments for Community-Acquired Pneumonia in Children: A Meta-analysis. Pediatrics 2023; 151(6): e2022060097. https://dx.doi.org/10.1542/peds.2022-060097.
11. Gulani A, Sachdev HPS, Qazi SA. Efficacy of short course (<4 days) of antibiotics for treatment of acute otitis media in children: A systematic review of randomized controlled trials. Indian Pediatr 2010; 47(1): 74-87. https://dx.doi.org/10.1007/s13312-010-0010-9.
12. Haider BA, Saeed MA, Bhutta ZA. Short-course versus long-course antibiotic therapy for nonsevere community-acquired pneumonia in children aged 2 months to 59 months. Cochrane Database Syst Rev 2008; (2): CD005976. https://dx.doi.org/10.1002/14651858.CD005976.pub2.
13. Hanretty AM, Gallagher JC. Shortened Courses of Antibiotics for Bacterial Infections: A Systematic Review of Randomized Controlled Trials. Pharmacotherapy 2018; 38(6): 674-687. https://dx.doi.org/10.1002/phar.2118.
14. Kim MS, Kim JH, Ryu S et al. Comparative efficacy and optimal duration of first-line antibiotic regimens for acute otitis media in children and adolescents: a systematic review and network meta-analysis of 89 randomized clinical trials. World J Pediatr 2023. https://dx.doi.org/10.1007/s12519-023-00716-8.
15. Kozyrskyj AL, Hildes-Ripstein GE, Longstaffe SEA et al. Treatment of acute otitis media with a shortened course of antibiotics: A meta-analysis. JAMA 1998; 279(21): 1736-1742. https://dx.doi.org/10.1001/jama.279.21.1736.
16. Kuitunen I, Jaaskelainen J, Korppi M, Renko M. Antibiotic Treatment Duration for Community-Acquired Pneumonia in Outpatient Children in High-Income Countries-A Systematic Review and Meta-Analysis. Clin Infect Dis 2023; 76(3): E1123-E1128. https://dx.doi.org/10.1093/cid/ciac374.
17. Lan SH, Lai CC, Chang SP et al. Five-day antibiotic treatment for community-acquired bacterial pneumonia: A systematic review and meta-analysis of randomized controlled trials. Journal of Global Antimicrobial Resistance 2020; 23: 94-99. https://dx.doi.org/10.1016/j.jgar.2020.08.005.
18. Li JZ, Winston LG, Moore DH, Bent S. Efficacy of Short-Course Antibiotic Regimens for Community-Acquired Pneumonia: A Meta-analysis. Am J Med 2007; 120(9): 783-790. https://dx.doi.org/10.1016/j.amjmed.2007.04.023.
19. Li Q, Zhou Q, Florez ID et al. Short-Course vs Long-Course Antibiotic Therapy for Children With Nonsevere Community-Acquired Pneumonia: A Systematic Review and Meta-analysis. JAMA Pediatrics 2022; 176(12): 1199-1207. https://dx.doi.org/10.1001/jamapediatrics.2022.4123.
20. Lopez-Alcalde J, Rodriguez-Barrientos R, Redondo-Sanchez J et al. Short-course versus long-course therapy of the same antibiotic for community-acquired pneumonia in adolescent and adult outpatients. Cochrane Database Syst Rev 2018; 2018(9): CD009070. https://dx.doi.org/10.1002/14651858.CD009070.pub2.
21. Lyon E, Olarte L. Community-acquired bacterial pneumonia in children: an update on antibiotic duration and immunization strategies. Curr Opin Pediatr 2024. https://dx.doi.org/10.1097/mop.0000000000001325.
22. Marques IR, Calvi IP, Cruz SA et al. Shorter versus longer duration of Amoxicillin-based treatment for pediatric patients with community-acquired pneumonia: a systematic review and meta-analysis. Eur J Pediatr 2022; 181(11): 3795-3804. https://dx.doi.org/10.1007/s00431-022-04603-8.
23. Moller Gundersen K, Nygaard Jensen J, Bjerrum L, Hansen MP. Short-course vs long-course antibiotic treatment for community-acquired pneumonia: A literature review. Basic Clin Pharmacol Toxicol 2019; 124(5): 550-559. https://dx.doi.org/10.1111/bcpt.13205.
24. Pichichero ME, Cohen R. Shortened course of antibiotic therapy for acute otitis media, sinusitis and tonsillopharyngitis. Pediatr Infect Dis J 1997; 16(7): 680‐695. https://dx.doi.org/10.1097/00006454-199707000-00011.
25. Schwarz EP. Update: amoxicillin for children with pneumonia Is a short course as effective as a long one? Geneesmiddelenbulletin 2023; 57(7): e2023.
26. Shaikh N, Hoberman A, Paradise JL et al. Association Between Nasopharyngeal Colonization and Clinical Outcome in Children With Acute Otitis Media. Pediatr Infect Dis J 2023; 42(8): e274-e277. https://dx.doi.org/10.1097/inf.0000000000003956.
27. Slawson DC. Amoxicillin for Children With CAP: Low-Dose for Three Days Is Noninferior to High-Dose for Seven Days. Am Fam Physician 2022; 105(5): 552C.
28. Sutijono D, Hom J, Zehtabchi S. Efficacy of 3-day versus 5-day antibiotic therapy for clinically diagnosed nonsevere pneumonia in children from developing countries. Eur J Emerg Med 2011; 18(5): 244-250. https://dx.doi.org/10.1097/MEJ.0b013e328344fd90.
29. Tansarli GS, Mylonakis E. Systematic review and meta-analysis of the efficacy of short-course antibiotic treatments for community-acquired pneumonia in adults. Antimicrob Agents Chemother 2018; 62(9). https://dx.doi.org/10.1128/aac.00635-18.
30. Venekamp RP, Schilder AGM. Clinical failure is more common in young children with acute otitis media who receive a short course of antibiotics compared with standard duration. Evid Based Med 2017; 22(3): 100. https://dx.doi.org/10.1136/ebmed-2017-110697.

Language not relevant (n=1)

1. Strachunsky L, Zharkova L, Kvirkveliia M et al. Treatment of outpatient chidlhood pneumonia by short course of azithromycin (results of randomized trial). Pediatriia 1997: 91‐96.

No full text publication available (n=2)

1. Kartasasmita C, Saha S, Short Course Cotrimoxazole Study Group. Consultative meeting to review evidence and research priorities in the management of acute respiratory infections (ARI) [unveröffentlicht]. 2022.
2. Lupison SP, Medalla FM, Miguel CA et al. A randomised, placebo controlled trial of short course cotrimoxazole for the treatment of pneumonia in Filipino children. Philippine Journal of Microbiology and Infectious Diseases 1999; 28(1): 15-20.

**Studies on Community-acquired pneumonia (CAP) (n=10)**

1. MASCOT. Clinical efficacy of 3 days versus 5 days of oral amoxicillin for treatment of childhood pneumonia: a multicentre double-blind trial. Lancet 2002; 360(9336): 835-841. <https://dx.doi.org/10.1016/s0140-6736(02)09994-4>.
2. ISCAP Study Group, Agarwal G, Awasthi S et al. Three day versus five day treatment with amoxicillin for non-severe pneumonia in young children: a multicentre randomised controlled trial. BMJ 2004; 328(7443): 791. <https://dx.doi.org/10.1136/bmj.38049.490255.DE>.
3. Greenberg D, Givon-Lavi N, Sadaka Y et al. Short-course antibiotic treatment for community-acquired alveolar pneumonia in ambulatory children: a double-blind, randomized, placebo-controlled trial. Pediatr Infect Dis J 2014; 33(2): 136-142. <https://dx.doi.org/10.1097/inf.0000000000000023>.
4. Ginsburg AS, Mvalo T, Nkwopara E et al. Amoxicillin for 3 or 5 Days for Chest-Indrawing Pneumonia in Malawian Children. N Engl J Med 2020; 383(1): 13-23. <https://dx.doi.org/10.1056/NEJMoa1912400>.
5. Barratt S, Bielicki JA, Dunn D et al. Amoxicillin duration and dose for community-acquired pneumonia in children: the CAP-IT factorial non-inferiority RCT. Health Technology Assessment (Winchester, England) 2021; 25(60): 1-72. <https://dx.doi.org/10.3310/hta25600>.
6. Bielicki JA, Stohr W, Barratt S et al. Effect of Amoxicillin Dose and Treatment Duration on the Need for Antibiotic Re-treatment in Children With Community-Acquired Pneumonia: The CAP-IT Randomized Clinical Trial. JAMA 2021; 326(17): 1713-1724. <https://dx.doi.org/10.1001/jama.2021.17843>.
7. Pernica JM, Harman S, Kam AJ et al. Short-Course Antimicrobial Therapy for Pediatric Community-Acquired Pneumonia: The SAFER Randomized Clinical Trial. JAMA Pediatrics 2021; 175(5): 475-482. <https://dx.doi.org/10.1001/jamapediatrics.2020.6735>.
8. Ginsburg AS, May S. Analysis of serious adverse events in a pediatric community-acquired pneumonia randomized clinical trial in Malawi. Sci Rep 2022; 12(1): 3538. <https://dx.doi.org/10.1038/s41598-022-07582-w>.
9. Pettigrew MM, Kwon J, Gent JF et al. Comparison of the Respiratory Resistomes and Microbiota in Children Receiving Short versus Standard Course Treatment for Community-Acquired Pneumonia. mBio 2022; 13(2): e0019522. <https://dx.doi.org/10.1128/mbio.00195-22>.
10. Williams DJ, Creech CB, Walter EB et al. Short- vs Standard-Course Outpatient Antibiotic Therapy for Community-Acquired Pneumonia in Children: The SCOUT-CAP Randomized Clinical Trial. JAMA Pediatrics 2022; 176(3): 253-261. <https://dx.doi.org/10.1001/jamapediatrics.2021.5547>.
